# Behavioural and neural markers of tactile sensory processing in infants at elevated likelihood of autism spectrum disorder and/or attention deficit hyperactivity disorder

**DOI:** 10.1186/s11689-020-09334-1

**Published:** 2021-01-04

**Authors:** Elena Serena Piccardi, Jannath Begum Ali, Emily J. H. Jones, Luke Mason, Tony Charman, Mark H. Johnson, Teodora Gliga, BASIS/STAARS Team, Mary Agyapong, Tessel Bazelmans, Leila Dafner, Mutluhan Ersoy, Amy Goodwin, Rianne Haartsen, Alexandra Hendry, Rebecca Holman, Sarah Kalwarowsky, Anna Kolesnik, Sarah Lloyd-Fox, Greg Pasco, Andrew Pickles, Laura Pirazzoli, Chloë Taylor

**Affiliations:** 1 Centre for Brain and Cognitive Development, Department of Psychological Sciences, Birkbeck, University of London, London, UK; 2 Institute of Psychiatry, Psychology & Neuroscience, Psychology Department, King’s College London, London, UK; 3 Department of Psychology, Cambridge University, Cambridge, UK; 4 Department of Psychology, University of East Anglia, Norwich, UK

**Keywords:** Autism spectrum disorder, Attention deficit hyperactivity disorder, Tactile sensory processing, Tactile sensory seeking, Repetition suppression, EEG, Alpha amplitude desynchronization, Infant sibling design

## Abstract

**Backgrounds:**

Atypicalities in tactile processing are reported in autism spectrum disorder (ASD) and attention deficit hyperactivity disorder (ADHD) but it remains unknown if they precede and associate with the traits of these disorders emerging in childhood. We investigated behavioural and neural markers of tactile sensory processing in infants at elevated likelihood of ASD and/or ADHD compared to infants at typical likelihood of the disorders. Further, we assessed the specificity of associations between infant markers and later ASD or ADHD traits.

**Methods:**

Ninety-one 10-month-old infants participated in the study (*n* = 44 infants at elevated likelihood of ASD; *n* = 20 infants at elevated likelihood of ADHD; *n* = 9 infants at elevated likelihood of ASD and ADHD; *n* = 18 infants at typical likelihood of the disorders). Behavioural and EEG responses to pairs of tactile stimuli were experimentally recorded and concurrent parental reports of tactile responsiveness were collected. ASD and ADHD traits were measured at 24 months through standardized assessment (ADOS-2) and parental report (ECBQ), respectively.

**Results:**

There was no effect of infants’ likelihood status on behavioural markers of tactile sensory processing. Conversely, increased ASD likelihood associated with reduced neural repetition suppression to tactile input. Reduced neural repetition suppression at 10 months significantly predicted ASD (but not ADHD) traits at 24 months across the entire sample. Elevated tactile sensory seeking at 10 months moderated the relationship between early reduced neural repetition suppression and later ASD traits.

**Conclusions:**

Reduced tactile neural repetition suppression is an early marker of later ASD traits in infants at elevated likelihood of ASD or ADHD, suggesting that a common pathway to later ASD traits exists despite different familial backgrounds. Elevated tactile sensory seeking may act as a protective factor, mitigating the relationship between early tactile neural repetition suppression and later ASD traits.

## Background

Autism spectrum disorder (ASD) and attention deficit hyperactivity disorder (ADHD) are heritable neurodevelopmental disorders emerging early in life. ASD affects up to 1.9% of the population [60], and core features of the conditions are social communication difficulties, restricted and repetitive behaviours and sensory atypicalities (DSM-5 [4];). ADHD affects up to 3.4% of the population [76] and core features of the condition are attentional control difficulties, hyperactivity and impulsivity (DSM-5 [4];). ASD and ADHD co-occur more often than expected based on their individual incidences, with co-occurrence rates ranging from 40 to 80% [5, 48]. Co-aggregation is reported in individuals and families [31]. Further, later-born siblings of children with ASD or ADHD appear to be more likely to develop the same disorder as their older sibling, but also the other disorder [63]. An aetiological link between ASD and ADHD is supported by twin and family studies [69, 83, 84], and correlated genetic variants are reported across the conditions [84, 94]. Thus, some common developmental mechanisms are proposed to underlie the emergence of ASD and ADHD but specific pathways have not been identified [45–47].

Evidence from prospective studies of infants at elevated likelihood of ASD or ADHD (i.e. by virtue of having a first-degree relative with the disorder) highlights similarities and differences in early markers of the two conditions. In particular, icommonalities are seen in early sensory markers [33, 45–47, 96]. For example, reduced habituation of EEG responses to repeated auditory tones in infancy associate with later ADHD [42] and ASD symptoms [53]. Further, atypicalities in tactile processing (i.e. tactile hyper/hyposensitivity and atypical tactile seeking) are documented by parental reports in both conditions [7, 29, 30, 97]. Motor atypicalities reported in the early development of ASD and ADHD (e.g. [27, 38, 44]) may be a consequence of common sensory vulnerabilities, given the tight link existing between the sensory and motor domains [30, 100]. Despite accumulating evidence that sensory-motor vulnerabilities manifest in the early development of both ASD and ADHD, no study has yet investigated the same sensory-motor markers as predictors of later ASD and/or ADHD traits. Investigating the specificity of early infant markers is essential to distinguish shared or distinct causal pathways and to understand the nature of the co-occurrence and the aetiology of these disorders.

Much research on early sensory processing within the neurodevelopmental disorder literature has focused on the visual or auditory modalities [9, 59], with no study yet assessing the potential mechanisms underlying early tactile atypicalities through controlled experimental designs or direct assessments of brain function. Filling this gap in knowledge is essential, given that (1) touch is the first sense to develop and the mean through which infants learn about the environment and themselves [13], (2) touch is the primary modality through which infants and caregivers communicate and interact [15, 26, 58], (3) difficulties in tactile processing dominate first-hand accounts from individuals with ASD [7, 35], and (4) many animal models of sensory atypicality in ASD focus on the tactile modality [18, 32, 40, 71].

### Tactile processing in ASD and ADHD

#### Behavioural markers

Different average responses to tactile stimulation are reported in young populations with ASD or ADHD relative to control participants ([31, 41, 62], and patterns of behavioural hyper/hyposensitivity to tactile stimulation are documented in the literature [16, 96]. Parent-reported, teacher-reported (e.g. Infant-Toddler Sensory Profile, *ITSP* [25];), examiner-reported or self-reported (e.g. Sensory Processing Scale, *SPS* [87];) measures indicate that behavioural hypersensitivity to tactile stimulation exists in children with ASD and persist through adulthood [7, 95, 97]. Cascio et al. [17] documented a pattern of behavioural hypersensitivity to tactile stimulation and lower self-reported judgement of tactile pleasantness in children with ASD, which associated with elevated severity of social symptoms. Further, in a retrospective study of children with ASD relying on parent-reported measures, Silva and Schalock [91] observed signs of allodynia (i.e. painful response to touch). While the majority of prior studies documented behavioural hypersensitivity to tactile stimulation in children with ASD, a few studies reported manifestations consistent with behavioural hyposensitivity to tactile input in the early development of the condition. For example, Baranek et al., [8], reported children with ASD to manifest behavioural hyposensitivity during a play-based observational assessment of various sensory modalities (including the tactile, visual and auditory modalities; Sensory Processing Assessment, Baranek: Sensory Processing Assessment for Young Children (SPA). Unpublished manuscript), which further predicted lower language scores. However, the authors assessed patterns of behavioural hyposensitivity across multiple modalities, rather than specifically addressing behavioural sensitivity to touch. More recently, Kadlaskar et al., [49] reported 12-month-old infants later diagnosed with ASD to manifest reduced orienting to caregiver touch, which was considered an indicator of early behavioural hyposensitivity to tactile stimulation and/or atypical social orienting. However, the authors also found that when infants with later ASD were already attending to touch-related locations prior to touch, they more frequently oriented away following caregiver’s touch, thus suggesting that behavioural hypersensitivity to tactile stimulation may be the predominant response when attention has already been allocated to the source of tactile input.

Research into tactile processing in ADHD is limited. Clinical investigations using self-reported, examiner-reported and parent-reported measures indicate that behavioural hyper/hyposensitivity to tactile stimulation co-exist in ADHD individuals and they might relate to different co-occurring symptoms. For example, Ghanizadeh [29, 30] reported that hypersensitivity associated with defiant oppositional symptoms, and hyposensitivity with separation anxiety symptoms in children with ADHD. Reduced discrimination of tactile input (e.g., temperature and pinprick discrimination) was documented in children with ADHD and their unaffected siblings, thus suggesting that hyposensitivity to tactile stimulation might be linked to familial liability for the disorder [86].

In summary, behavioural evidence suggests that tactile hypersensitivity mostly occurs in individuals with ASD. Tactile processing in ADHD remains understudied, although the current evidence points to co-occurring tactile hyper/hyposensitivity.

#### Neural markers

Neurophysiological studies on tactile processing in ASD have mainly investigated stimulus repetition effects [6, 70]. These paradigms allow quantification of two measures: (1) the effect of individual tactile stimulation on initial brain responses, henceforth *neural sensitivity*, and (2) the effect of repeating tactile stimulation, often manifested as a decrease in the response to the second stimulus with respect to the first stimulus, henceforth *neural repetition suppression*. Studies have generally documented reduced repetition suppression to tactile stimulation in ASD. Reduced neural repetition suppression to sequences of vibrotactile stimuli in the absence of stimulus-locked neural hypersensitivity was documented in a Fmr1 knock-out mouse model of ASD [40]. Increased blood-oxygen-level-dependent (BOLD) activation in the somatosensory cortex and amygdala was reported in response to mildly aversive tactile stimulation in young participants with ASD and attributed to reduced habituation of brain responses [36]. Controlled psychophysical studies have also suggested that reduced repetition suppression underlies the tactile performance of young participants with ASD.

For example, Puts et al., [80] reported no effect of an adapting (i.e. repeated) stimulus on tactile discrimination thresholds in children with ASD. The effect was replicated in a follow-up study and linked to reduced levels of the neurotransmitter GABA in the somatosensory cortex [78, 79].

Neurophysiological studies investigating tactile processing in ADHD are limited and document reduced neural repetition suppression of tactile stimulation and neural hyposensitivity. Neural hyposensitivity to non-painful current pulses, indexed by reduced somatosensory EEG alpha power desynchronization, was reported in adults with ADHD [23]. Increased perfusion in the post-central gyrus was observed in adults with ADHD and linked to inability to suppress incoming tactile input [52]. Controlled psychophysical studies reported higher detection thresholds and reduced repetition suppression in children with ADHD [78, 79]. Reduced levels of the neurotransmitter GABA were also reported in the somatosensory cortex of individuals with ADHD [25].

Overall, the reviewed evidence suggests that different neural responses to tactile stimulation occur in individuals with ASD or ADHD relative to control participants and these differences may result from atypical inhibitory function in GABA-mediated circuits. However, it remains unknown if these differences are present early in development and, if so, whether they associate with traits of ASD or ADHD emerging in childhood.

### The role of sensory seeking

Atypical responses to sensory stimulation are documented in the early development of ASD or ADHD but putative mechanisms linking these atypicalities to later traits remain unknown. In the tactile domain, early atypical responsiveness has been proposed to exacerbate later ASD symptomatology by triggering compensatory strategies aimed at minimizing tactile input [62].

Indeed, decreased sensory seeking is often reported in infants with later ASD [10, 67, 96], and some have proposed that it may mediate the impact of early sensory atypicality on later ASD traits [96, 101]. In the tactile domain, decreased seeking could represent a strategy to minimize tactile input (which may be experienced as distressing in the presence of elevated sensory responsiveness, [45, 46, 67]). However, reduced sensory seeking has not always been found to associate with elevated sensory responsiveness [10, 75]. Thus, rather than a mediator, sensory seeking could represent an independent but compounding factor in ASD. For example, it has been proposed that reduced sensory seeking in infants with later ASD reflects reduced capacity or motivation to explore, rather than a consequence of atypical sensory responsiveness [67]. Under this scenario, lower sensory seeking may increase the impact of sensory atypicalities by further limiting early opportunities to develop social skills and share communication.

### The current study

The goal of the current study was to investigate behavioural and neural markers of tactile sensory processing in 10-month-old infants at elevated likelihood of ASD or ADHD (i.e. by virtue of having a first-degree relative with a clinical diagnosis of ASD or ADHD) and infants at typical likelihood of the disorders. A tactile repetition suppression paradigm administering repeated pairs of vibrotactile stimuli (S1–S2) was used and coupled with the recording of EEG. We quantified behavioural markers by coding looking and moving behaviours before and after receiving the pair of tactile stimuli. We quantified neural markers by extracting the amplitude of EEG oscillations in the alpha range (6–10 Hz). The choice of analyzing the alpha rhythm (i.e. oscillations in the range of 8–12 Hz in adults and 6–10 Hz in infants) in the present study was motivated by three reasons. First, the EEG alpha rhythm has been specifically associated with GABAergic inhibitory modulation in the somatosensory cortex in animals [57] and humans [1, 88]. Thus, early differences in GABA-mediated inhibitory modulation in somatosensory regions should be reflected by differences in alpha amplitude desynchronization (i.e. alpha amplitude during the task as compared to alpha amplitude at baseline) over the somatosensory cortex. Secondly, while GABAergic inhibition has also been associated with other EEG frequency bands (e.g. gamma rhythm), these associations are not specific to somatosensory regions (and have mostly been reported in other sensory modalities, e.g. auditory modality [53];). Thirdly, while event-related potentials (ERPs) have most commonly been employed to quantify repetition suppression, mainly in the auditory modality [72], the literature on tactile ERPs in early development is scanty and no study has so far assessed ERPs in a tactile repetition suppression paradigm in infancy, thus limiting our ability to specify a priori testable predictions (e.g. in regard to the choice of ERP components to subject to statistical analysis). Furthermore, the specificity of ERPs is debated and the neurophysiological dynamics that give rise to ERPs are not well understood [19], thus providing limited opportunity for linking results to physiological mechanisms such as GABAergic inhibition. Finally, ERPs contain little information about the underlying EEG dynamics and task-related information can be lost in the process of ERP averaging (see [19], who provides an excellent demonstration that non-phase-locked dynamics are task-modulated but not observable in the ERPs).

Based on previous work on tactile processing in ASD and ADHD, we predicted observing an effect of ASD likelihood status on behavioural sensitivity, manifesting as elevated moving and reduced screen-directed looking (hypersensitivity) after receiving the tactile stimulation. Since atypical neural repetition suppression has been documented in ASD and ADHD, we predicted observing an effect of ASD and ADHD likelihood on neural response to repeated tactile input, manifesting as reduced suppression of alpha amplitude desynchronization to repeated tactile stimulation. We further predicted observing an effect of ADHD likelihood status on neural sensitivity, manifesting as reduced alpha amplitude desynchronization (neural hyposensitivity) to the first vibrotactile stimulus.

We assessed the longitudinal associations between early neural (and behavioural, see SM2) markers of tactile processing and later ASD traits (i.e. quantified through the Autism Diagnostic Observation Schedule, Second edition (ADOS-2) calibrated severity scores (CSS) at 24 months [56]; Q-CHAT at 24 months in SM2 [3];) or ADHD traits (i.e. quantified through the Early Childhood Behaviour Questionnaire (ECBQ) activity and inhibitory control sub-scales at 24 months [77];). Previous research indicates that these measures act as early predictors of later symptoms of ASD and ADHD, respectively. Shephard et al. [90] documented that higher 24-month ECBQ activity levels and inhibitory control predict higher mid-childhood hyperactivity/impulsivity and inattention but not ASD symptoms. Overall stability in ADOS CSS was also reported between the ages of 2 and 15 years [34]. Therefore, ADOS-2 CSS and ECBQ activity and inhibitory control were designated as 24-month outcome measures in the current study. We predicted reduced neural repetition suppression to longitudinally predict both ASD and ADHD traits. We further predicted reduced alpha amplitude desynchronization to the first vibrotactile stimulus (neural hyposensitivity) at 10 months to associate with higher activity level and lower inhibitory control at 24 months.

As a final step, we explored the role of tactile sensory seeking (i.e. quantified through the parent-reported Infant-Toddler Sensory Profile at 10 months, ITSP [24];) as a potential mediator or moderator of the association between early tactile atypicality and later ASD traits.

## Methods

### Recruitment approach

Participants were recruited for a longitudinal study running from 2013 to 2019. The recruitment and categorization approach adopted in the present study is the same employed by Begum et al., [11]. In particular, infants could be enrolled in the study if they either had a first-degree relative with ASD, a first-degree relative with diagnosed or probable ADHD or no first-degree relatives with either diagnosis. We defined the presence of ASD as a clinical diagnosis of ASD from a licensed clinician. We defined the presence of ADHD as a community clinical diagnosis of ADHD or a probable research diagnosis of ADHD. For those who report concerns of ADHD symptoms in the family where the parent or older sibling does not have a community clinical diagnosis of ADHD, screening questionnaires are used to examine the probable existence of ADHD (see SM1). This was implemented because co-occurring conditions are often underdiagnosed in children with ASD (e.g., [68]; see [99] for a review of co-occurrence rates), primarily because previously DSM-IV and ICD-10 did not allow a dual diagnosis of ASD and ADHD. Had we required a clinical diagnosis for an infant to be coded as “elevated likelihood of ADHD”, we would have risked under-identification in those families with a proband with an ASD diagnosis, significantly compromising the familial diagnosis elevated likelihood design we adopted for sampling. Further, we did not want to apply different criteria to those families with and without an older sibling with ASD. Thus, we adopted an additional screening process for ADHD in first-degree relatives. For siblings (aged less than 6 years), a shortened version of the Conners Early Childhood [20] form was used. For siblings (6 years or older), a shortened version of the Conners 3 was used. Thresholds for inclusion in the ADHD category were the presence of 6 ADHD symptoms on either the hyperactivity/impulsivity or inattention scale, and a positive score on the impairment scale. For parents, a shortened version of the Conners Adults ADHD Rating Scale (CAARS) was used. Thresholds for inclusion were the presence of 5 ADHD symptoms on either the hyperactivity/impulsivity or inattention scale as per updated DSM-5 guidelines.

In terms of use of the impairment scores, we adopted a reduced version of the Conners EC and Conners 3 for individuals under 18 and the CAARS for individuals aged 18+ years. The Conners EC and Conners 3 included questions regarding impairment, as such we also included these questions in our screening forms. In comparison, the CAARS (adult questionnaire) did not include questions regarding impairment. In order to maintain consistency of measure, we did not adapt the CAARS to add impairment questions. Of note, at initial contact with participants, parents were asked if there were any diagnoses of ADHD in the immediate family or if they had any concerns about ADHD. It is only if parents reported concerns that the screening process took place. This categorization protocol is very similar to that adapted by other labs using the prospective longitudinal study model in infants at elevated likelihood of ADHD (see [64]).

Each infant in the study was assigned a rating for elevated likelihood of ASD and ADHD. A rating of 1 for ASD indicated the presence of ASD in a parent or older sibling; a rating of 1 for ADHD indicated that presence of ADHD in a parent or older sibling; and a rating of 0 for either category indicated no *confirmed* presence of the relevant condition. Thus, infants at elevated likelihood of ASD (EL-ASD), infants at elevated likelihood of ADHD (EL-ADHD), infants at elevated likelihood of ASD and ADHD (EL-ASD+ADHD) and infants at typical likelihood of the conditions (TL) participated to this research. TL infants had at least one older sibling with typical development and no first-degree relatives with a diagnosis of ASD or ADHD. These infants were recruited from a volunteer database at the Centre for Brain and Cognitive Development, Birkbeck, University of London. All infants were born full-term (gestational age 38–42 weeks). At the time of enrolment, none of the infants had a known medical or developmental condition. Informed written consent was provided by the parent(s) prior to the commencement of the study. Infants were tested if awake and in an alert state. The experimental protocol was approved by the National Research Ethics Service and the Research Ethics Committee of the Department of Psychological Sciences, Birkbeck, University of London and the Psychology Department, King’s College London. Families were reimbursed expenses for travel, subsistence and overnight stay if required. Infants were given a certificate and t-shirt after each visit.

### Participants

One hundred and fifty-two 10-month-old infants participated in the study: 79 EL-ASD infants, 27 EL-ADHD infants, 21 EL-ASD+ADHD infants and 25 TL infants, with no family history of the disorders. Of these, 61 infants were tested but not included in the final sample because of low tolerance of the EEG net (*n* = 8), fussiness/excessive movement artefacts (*n* = 38) and equipment failure (*n* = 15). One infant contributed EEG data but was not included in the behavioural analyses due to missing video recording. Accordingly, EEG data was contributed by 91 infants (90 infants contributed behavioural data): 44 EL-ASD infants, 20 EL-ADHD infants, 9 EL-ASD+ADHD infants and 18 TL infants. For descriptive statistics see Tables 1 and 2. There was no significant effect of likelihood status on participants’ attrition rate, *χ*^2^(3) = 6.9, *p* = .075.

**Table 1:** Detailed characterization of behavioural measures at the 10- and 24-month assessments for EL-ASD, EL-ADHD, EL-ASD+ADHD and TL participants who contributed to the EEG analyses

	EL-ASD	EL-ADHD	EL-ASD+ADHD	TL	*p* values
10-month visit					
Age in days	318.65 (13.42)	326.55 (29.76)	316.56 (14.05)	323.22 (16.77)	.378 (ns)
MSEL ELC	86.47 (14.39)	86.05 (17.09)	80.78 (15.82)	91.11 (9.65)	.359 (ns)
MSEL GM	37.67 (8.51)	39.75 (9.92)	33.56 (9.81)	33.11 (9.87)	.102 (ns)
MSEL FM	50.14 (11.22)	53.65 (15.26)	46.55 (13.65)	50.78 (8.09)	.499 (ns)
MSEL VR	48.56 (9.09)	47.10 (10.46)	48.00 (8.29)	50.61 (5.75)	.669 (ns)
MSEL RL	36.30 (10.03)	34.65 (10.37)	34.11 (10.87)	41.05 (8.05)	.174 (ns)
MSEL EL	36.35 (13.05)	34.95 (13.22)	30.22 (12.97)	39.11 (9.86)	.367 (ns)
*N* (% boys)	43 (43.2)	20 (60)	9 (55.6)	18 (50)	
ITSP Tactile Seeking	2.40 (0.99)_a_	1.90 (0.54)	2.33 (0.97)	1.81 (0.39)	.003*
24-month visit					
Age in days	777.00 (19.66)	771.12 (40.38)	755.57 (19.66)	764.40 (43.63)	.610 (ns)
MSEL ELC	101.87 (20.87)_a_	104.76 (21.61)	92.86 (18.08)_a_	120.00 (15.53)	.011*
MSEL GM	N/A	N/A	N/A	N/A	
MSEL FM	50.95 (10.39)_a_	50.82 (11.90)	49.43 (12.15)	61.20 (10.72)	.015*
MSEL VR	50.20 (13.27)_a_	55.53 (11.86)	43.86 (8.80)_a_	63.67 (8.37)	.001**
MSEL RL	51.41 (14.11)	50.34 (13.44)	47.43 (9.13)	57.87 (7.43)	.217 (ns)
MSEL EL	49.47 (15.72)	52.23 (15.21)	44.28 (11.46)	58.00 (12.79)	.159 (ns)
*N* (% boys)	38 (42.1)	17 (58.8)	7 (57.1)	15 (40)	
ADOS-2 CSS	2.97 (2.26)_a_	2.65 (1.97)	3.14 (2.03)_a_	1.40 (0.63)	.040*
ECBQ inhibitory control	3.74 (1.29)	3.84 (0.97)	3.22 (1.61)	4.31 (0.92)	.250 (ns)
ECBQ activity	4.54 (0.84)	4.88 (1.04)	5.11 (0.74)	4.62 (0.72)	.325 (ns)
Q-CHAT	24.37 (12.17)	28.00 (10.96)	31.08 (15.19)	21.05 (3.82)	.202 (ns)

*p < .05

** *p* ≤ .001

_a_Significant differences with the TL group

*M* (*SD*) reported for age in days*, MSEL ELC* (Mullen Scales for Early Learning Early Composite Score), *MSEL GM* (Mullen Scales for Early Learning Gross Motor Score), *MSEL FM* (Mullen Scales for Early Learning Fine Motor Score), *MSEL VR* (Mullen Scales for Early Learning Visual reception Score), *MSEL RL* (Mullen Scales for Early Learning Receptive Language Score), *MSEL EL* (Mullen Scales for Early Learning Expressive Language), *ITSP Tactile Seeking* (tactile sensory seeking average score of the Infant-Toddler Sensory Profile), *ADOS-2 CSS* (ADOS-2 Calibrated Severity Scores), *ECBQ inhibitory control* (inhibitory control subscale of the Early Childhood Behaviour Questionnaire), *ECBQ activity* (activity subscale of the Early Childhood Behaviour Questionnaire), *Q-CHAT* (Quantitative Checklist for Autism in Toddlers)

**Table 2:** Number of EL-ASD, EL-ADHD, EL-ASD+ADHD and TL infants included and excluded from the 10-month EEG analyses (i.e. due to contributing less than 10 artifact-free trials) and number of trials presented and retained for each group

Participants (*n*)	EL-ASD	EL-ADHD	EL-ASD+ADHD	TL	*p* values
Included	44	20	9	18	.075 (ns)
Excluded	19	4	7	7	.318 (ns)
Trials (*n*)	EL-ASD	EL-ADHD	EL-ASD+ADHD	TL	*p* values
Presented	35	36	37	35	.570 (ns)
Retained	17	16	18	16	.865 (ns)

### Stimuli

**Fig. 1:**
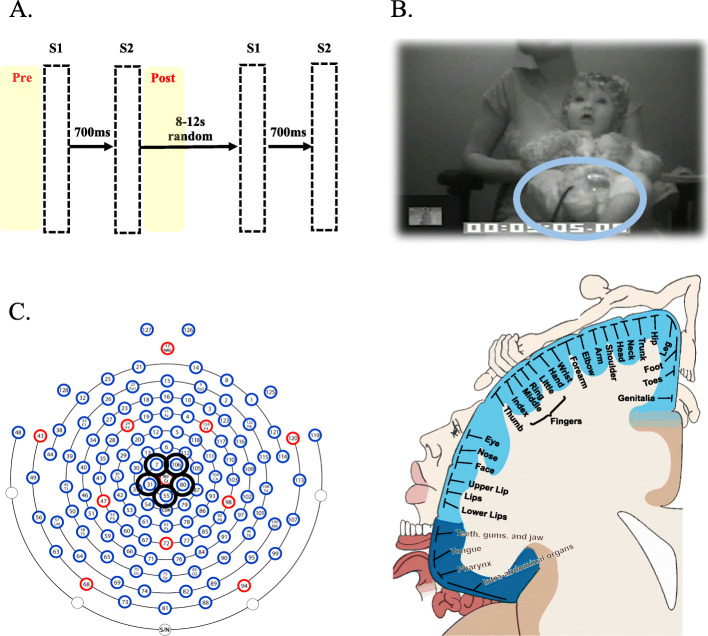
a Representation of the sequence of events in the tactile repetition suppression paradigm. Pairs of 200-ms-long vibrotactile stimuli were delivered to the infants’ feet with a 700-ms ISI within the pair and 8–12 s ISI between the pairs. Pre-stimulus and post-stimulus phases (4 s each) are highlighted in yellow. **b** High-density EEG was recorded while vibrotactile stimuli were delivered to the infants’ feet through custom-made tactors (the light blue circle indicates the location of one tactor). **c** Hydrocel-Geodesic Sensor Net montage displaying the central somatosensory pool of electrodes (black circle) used for quantifying α desynchronization (6–10 Hz) to vibrotactile stimulation. The pool corresponded spatially to the somatotopic representation of the human feet.

Vibrotactile stimuli were delivered by two custom-built voice coil tactors driven by a 220-Hz sine wave and controlled by a custom MATLAB® script. The choice of a 220-Hz sine wave as a tactile stimulus was based on prior literature investigating tactile perception in early typical development [2]. The tactors were placed in direct contact with the bare soles of the infant’s feet, securing them with cohesive bandage. A repetition suppression paradigm was used: pairs of 200-ms stimuli (S1–S2) were simultaneously delivered to both feet, with 700-ms inter-stimulus interval (ISI) (constant) within the pair and 8–12 s ISI (random) between the pairs (Fig. 1a). Thirty-eight pairs of vibrotactile stimuli were administered during two blocks lasting 4 min each, while infants underwent EEG. A 2-min interval corresponded to the end of the first block and beginning of the second block. An animated cartoon with no language component was presented throughout the session (*Fantasia* by Walt Disney) and served two functions: to distract infants’ attention away from the tactile stimulation and to mask the sound produced by the tactors themselves. Total experiment duration was 10 min but the experimenter could interrupt the session earlier in case of infant’s fussiness or if requested by the parent.

### Apparatus and procedure

Testing took place in a dimly illuminated room. Infants were seated on a parent’s lap, 60 cm from a screen (27 inches; width 59.77 cm, height 33.62 cm) and were allowed to use a pacifier. The sequence and timing of stimulus presentation were controlled using MATLAB®. High-density EEG was collected using 124 channels of a 128-channel HydroCel-Geodesic Sensor Net connected to a NetAmps 400 amplifier (Electrical Geodesic, Eugene, OR) and referenced on-line to the vertex (Cz). Signals were sampled at 500 Hz. A video camera situated below the screen used for stimulus presentation recorded the infants’ bodily and facial behaviour (Fig. 1b). This information was used for online monitoring of infants’ performance and offline behavioural coding.

### Behavioural assessment scales

The Mullen Scales of Early Learning [66] were administered at the 10 and 24-month visits in the standardized format. The 10-month Mullen data was collected for 90 out of 91 infants contributing to the EEG analyses. The 10-month ITSP was returned for 78 out 91 participants contributing to the EEG analyses. At 24 months, 12 participants dropped-out from the longitudinal study. Thus, at this visit, Mullen data was collected for 77 participants and ADOS-2 assessment was performed for 79 out of 91 infants contributing to the EEG analyses. The 24-month Q-CHAT was returned for 74 participants (see SM2 for analyses on this measure). The 24-month ECBQ was returned for 71 participants. Detailed characterization of each measure for participants contributing to the EEG analyses is reported in Table 1. Full characterization is reported in SM4 Table 1. We also report in SM details on how scores indexing the sensory seeking quadrant within the tactile domain of the ITSP were computed, alongside assessment of their internal consistency and composite reliability (SM1) and investigation of the effect of likelihood status on this measure (SM2).

### Infants’ behaviour coding

Infants’ bodily and facial behaviour was scored with a computerized frame-by-frame coding system (25 frames/second—EGI Movie Player, Electrical Geodesic). The category of body movement included any head, upper and lower limbs or feet movements. The category of facial behaviour included only screen-directed looking. Looking and movement were scored using a binary coding procedure (i.e. looking = 1; not looking = 0; moving = 1; not moving = 0) during the “pre-stimulus phase” (4 s before S1) and the “post-stimulus phase” (4 s after S2) (Fig. 1a). The binary codes of looking vs. not looking and moving vs. not moving were calculated based on whether looking or movement occurred/did not occur during the pre-stimulus and post-stimulus phases. No coding was performed during the 700-ms ISI because the interval was too short to observe changes in infants’ looking or body movement. A second observer independently coded a random 40% of video files (i.e. 36 infants). Both coders were blind to infants’ likelihood status. Conversely, coders were not blind to trial period (i.e. “pre-stimulus phase” and “post-stimulus phase”).

An interrater reliability analysis using intra-class correlation (ICC; absolute agreement type, average measures) indicated high agreement for looking behaviours during the pre-stimulus phase, ICC = .996, 95% CI [.992, .998], *p* < .001; for looking behaviours during the post-stimulus phase; ICC = .998, 95% CI [.996, .998], *p* < .001; for body movement behaviours during the pre-stimulus phase, ICC = .994, 95% CI [.989, .997], *p* < .001; for body movement behaviours during the post-stimulus phase, ICC = .997, 95% CI [.994, .998], *p* < .001.

### EEG recording and analysis

EEG data was pre-processed offline using Net Station (Electrical Geodesic). Continuous EEG was filtered using a 0.3–40-Hz band-pass filter. The EEG signal was segmented from 200 ms prior to S1 onset through 1800 ms after S1 onset. Automated artifact detection was applied to the segmented data to detect individual epochs that showed > 200-μV voltage changes within the segment period. EEG recordings were visually inspected, and individual channels within segments were eliminated from the analysis if artifacts occurred. Segments in which > 15% of the channels (18 channels) were marked as bad were excluded from the analysis. For the remaining trials, spherical spline interpolation was conducted to replace data for bad channels using the five closest electrodes. Infants were excluded from the analysis if they had less than 10 artifact-free segments (see Table 2).

### Time-frequency analysis of EEG

**Fig. 2:**
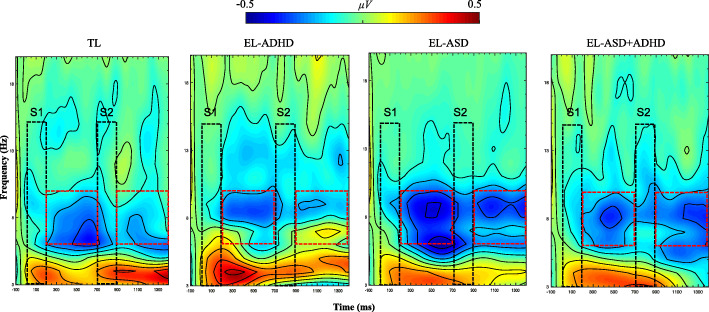
Time-frequency plots illustrating the amplitude of α (6–10 Hz) oscillations time-locked to S1 and S2 offset for each participant group (TL = infants at typical likelihood of ASD or ADHD; EL-ADHD = infants at elevated likelihood of ADHD; EL-ASD = infants at elevated likelihood of ASD; EL-ASD+ADHD = infants at elevated likelihood of ASD and ADHD). Black dotted rectangles indicate the first and second vibrotactile stimulations. Red dotted squares indicate the 500-ms-long time-windows post-stimulus offset selected for statistical analysis. Amplitude scale is − 0.5, 0.5 μv

Time-frequency decomposition was used to quantify oscillatory alpha amplitude desynchronization to tactile stimulation (i.e. 6–10-Hz alpha amplitude during the task as compared to alpha amplitude at baseline). Artifact-free segments were imported into MATLAB® using EEGLAB (v. 13.4.3b) and re-referenced to the average reference. The collection of scripts *WTools* (see [73]; available upon request) was used for spectral decomposition, employing complex Morlet wavelets for the frequencies 3–20-Hz (1-Hz resolution). A continuous wavelet transformation of all segments by means of convolution with each wavelet was performed, and the absolute value of the results was extracted. One hundred milliseconds of data was cut at segment ends to remove the distortion due to convolution. The amplitude of the 100-ms pre-stimulus window was used as a baseline and subtracted from the whole epoch at each frequency. Individual epochs were averaged for each participant. Inspection of the time-frequency plots revealed that 6–10-Hz alpha amplitude desynchronization occurred at S1 and S2 offset over the central scalp site. Based on previous literature [21] and on visual inspection of both the grand-averaged and individual time-frequency plots, channels (CH) 7, 31, 55, 80, and 106 (Fig. 1c) were selected and the average 6–10-Hz alpha desynchronization oscillatory amplitude extracted for two 500-ms-long time windows time-locked to S1 and S2 offset, respectively (Fig. 2). Two alpha amplitude desynchronization measures were computed: S1 alpha amplitude desynchronization (indexing neural sensitivity to the first vibrotactile stimulus) and S2–S1 alpha amplitude desynchronization or tactile suppression index (TSI; indexing neural repetition suppression of tactile stimulation).

### Analytical strategy

Statistical analyses were conducted in SPSS v23 [43]. Likelihood status was dummy coded, and a factorial approach was used to test for the main effect of ASD, ADHD and the interaction between these factors on behavioural and EEG markers of tactile processing. The likelihood factor was computed as follows: EL-ASD infants were assigned a “1” for ASD likelihood and a “0” for ADHD likelihood (1 0), EL-ADHD infants were assigned a “0” for ASD likelihood and a “1” for ADHD likelihood (0 1), EL-ASD+ADHD infants were assigned a “1” for ASD likelihood and a “1” for ADHD likelihood (1 1) and TL infants were assigned a “0” for ASD likelihood and a “0” for ADHD likelihood (0 0). This approach was taken to examine any additive/protective effects of having an elevated likelihood of both disorders. For tables and figures, infants were split into four groups: EL-ASD, EL-ADHD, EL-ASD+ADHD and TL.

Prior to performing any inferential statistical analyses, the variables were assessed for normality. Where significant violations of normality existed, data was normally transformed (i.e. details on normality violations and transformations are reported in the results section).

First, we assessed the effect of likelihood status on behavioural markers of sensitivity to tactile stimulation during the experiment. We ran separate repeated measures ANOVAs with stimulation (two levels: pre-stimulus and post-stimulus) as within-subject factor and screen-directed looking or body movement occurring during each phase as dependent variables, respectively. In SM2, we reported results from the same analyses conducted on an extended sample of participants, which included those who contributed EEG data and those who were excluded from the EEG analyses due to fussiness/movement artifacts. Further, in SM2, we reported results from analyses assessing the effect of likelihood status on behavioural markers of sensitivity to tactile stimulation assessed through parental reports (i.e. ITSP [24];). We ran separate univariate ANOVAs with tactile sensory sensitivity and low registration as dependent variables, respectively. We further conducted these analyses on the extended participant sample. Furthermore, we assessed in SM2 the consistency between behavioural markers of sensitivity to tactile stimulation assessed during the EEG task and assessed through parental reports (i.e. ITSP [24];) through sets of Pearson correlations.

Secondly, we assessed the effect of likelihood status on neural markers of tactile sensory processing. We ran separate univariate ANOVAs with sensitivity to tactile stimulation (i.e. alpha amplitude desynchronization to the first vibrotactile stimulus, S1) and neural repetition suppression (i.e. TSI, S2–S1) as dependent variables, respectively.

Thirdly, we examined the longitudinal associations between neural markers (and behavioural markers, in SM2) and later ASD or ADHD traits with a set of hierarchical linear regressions for normally distributed outcome measures or Spearman correlations for non-normally distributed outcome measures. When significant associations between predictor and one outcome variable existed, we further investigated the potential moderating effect of the likelihood factors on these associations.

Finally, we explored the role of tactile sensory seeking as a mediator or moderator of the relationship between early tactile atypicality and later ASD traits. The mediation and moderation analyses were conducted using PROCESS macro in SPSS [39]. Significant moderation effects were further explored through spotlight and floodlight analyses [93]. A simple slop plot for illustrating results of the spotlight analysis and a Johnson-Neyman plot for illustrating results of the floodlight analysis were generated with the workbook CAHOST [14]. In SM2, we further investigated the potential mediating or moderating role of tactile sensory avoiding (this analysis was conducted following the same pipeline used for assessing the mediating/moderating effect of tactile sensory seeking; see the “Additional analyses” section for further details).

## Results

### Behavioural markers

#### Screen-directed looking

A main effect of stimulation (pre- vs. post-stimulus phase) emerged, F(1,86) = 16.54, *p* < .001, *η*^2^ = .161, indicating looking away from the screen after receiving the tactile stimulation. There was no significant interaction between stimulation and ASD likelihood status, F(1,86) = 0.82, *p* = .776, *η*^2^ = .001, or between stimulation and ADHD likelihood status, F(1,86) = 1.97, *p* = .164, *η*^2^ = .022. There was also no significant three-way interaction between stimulation, ASD and ADHD likelihood status, F(1,86) = 1.006, *p* = .319, *η*^2^ = .012, see SM3 Fig. 1a.

#### Body movement

A main effect of stimulation (pre- vs. post-stimulus phase) emerged, F(1,86) = 29.87, *p* < .001, *η*^2^ = .258, indicating increased movement after receiving the tactile stimulation. There was, however, no significant interaction between stimulation and ASD likelihood status, F(1,86) = .001, *p* = .995, *η*^2^ = .000, or between stimulation and ADHD likelihood status, F(1,86) = 3.35, *p* = .071, *η*^2^ = .037. There was also no significant three-way interaction between stimulation, ASD and ADHD likelihood status, F(1,86) = .081, *p* = .776, *η*^2^ = .001, see SM3 Fig. 1b.

### Neural markers

#### Neural sensitivity (S1)

**Fig. 3:**
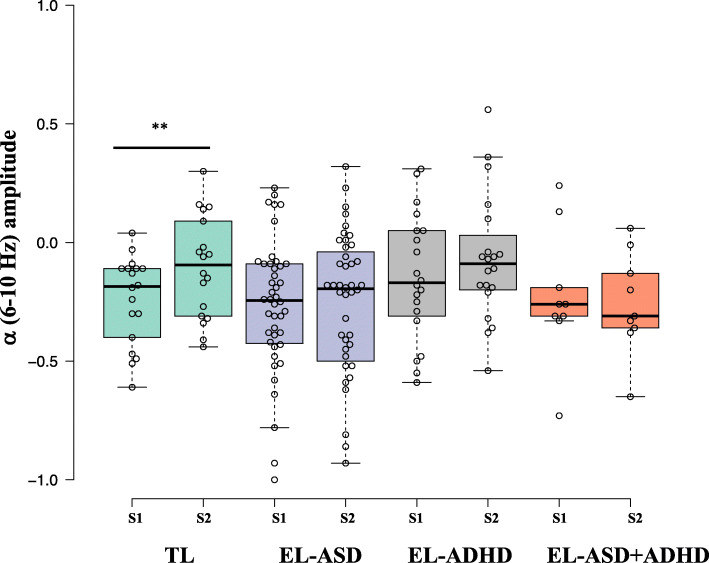
Boxplots illustrating the amplitude of α (6–10 Hz) oscillations time-locked to S1 and S2 offset for each participant group (green = infants at typical likelihood of ASD or ADHD; violet = infants at elevated likelihood of ASD; grey = infants at elevated likelihood of ADHD; orange = infants at elevated likelihood of ASD and ADHD). A significant reduction in α desynchronization with repeated tactile stimulation occurred only in TL infants. **p < .001

There was no significant main effect of ASD likelihood status, F(1,87) = .803, *p* = .373, *η*^2^ = .009, or ADHD likelihood status, F(1,87) = 1.267, *p* = .263, *η*^2^ = .014, and no significant interaction between ASD and ADHD likelihood status, F(1,87) = .034, *p* = .854, *η*^2^ = .000, see Figs. 2 and 3.

#### Neural repetition suppression (TSI: S2–S1)

**Fig. 4:**
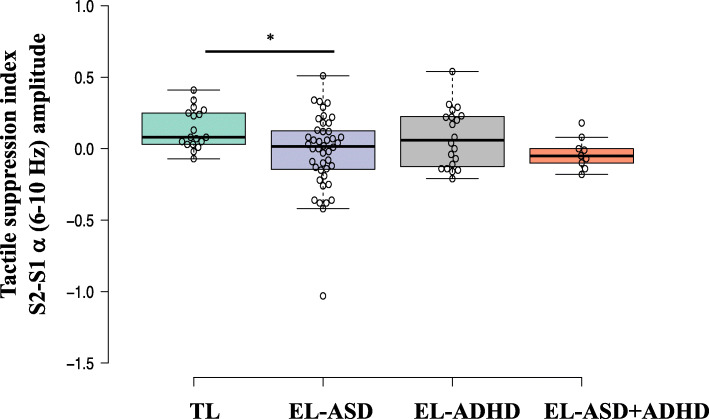
Boxplot illustrating the tactile suppression index, α (6–10 Hz), for each participant group (green = infants at typical likelihood of ASD or ADHD; violet = infants at elevated likelihood of ASD; grey = infants at elevated likelihood of ADHD; orange = infants at elevated likelihood of ASD and ADHD). *p < .05

Infants with an elevated ASD likelihood manifested reduced neural repetition suppression to tactile stimulation F(1,87) = 6.089, *p* = .016, *η*^2^ = .065. There was no significant main effect of ADHD likelihood status, F(1,87) = .366, *p* = .547, *η*^2^ = .004. Further, there was no significant interaction between ASD and ADHD likelihood status, F(1,87) = .229, *p* = .634, *η*^2^ = .003, see Fig. 4.

### Associations between neural markers and later ASD or ADHD traits

We report below the associations between neural markers of tactile sensory processing and later ASD or ADHD traits. In SM2, we report the associations between the same neural measures and parental reports of ASD traits (i.e. quantified through the Q-CHAT at 24 months) and general development (i.e. quantified through Mullen Scales at 10 and 24 months).

#### Associations with ASD traits at 24 months

ADOS-2 CSS significantly violated normality assumptions (Shapiro-Wilk, *p* < .001; Skewness = 1.471, SE = .218; Kurtosis = 1.571, SE = .433) and were log-transformed prior to the analyses.

The hierarchical linear regression with S1 alpha amplitude desynchronization as predictor and ADOS-2 CSS (log) as outcome was not statistically significant, F(1,77) = .317, *p* = .575, *R*^2^_adj_ = .004. The result did not change when ECBQ activity was partialled out, F(2,66) = .245, *p* = .783, *R*^2^_adj_ = .000, 95% CI for B [− .147, .235] and when ECBQ inhibitory control was partialled out, F(2,65) = 1.382, *p* = .258, *R*^2^_adj_ = .011, 95% CI for B [− .249, .030].

**Fig. 5:**
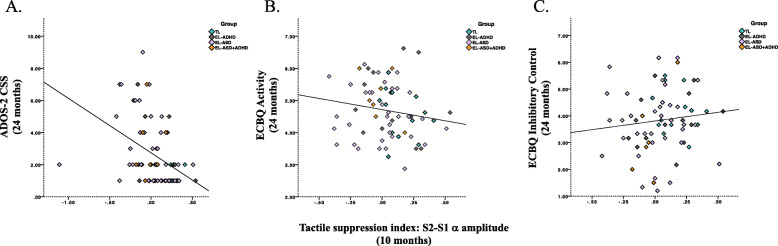
Scatterplots illustrating the associations between tactile suppression index (S2–S1 α amplitude) at 10 months and **a** ADOS-2 CSS at 24 months (*p* < .001), **b** ECBQ activity at 24 months (*p* = ns) and **c** ECBQ inhibitory control at 24 months (*p* = ns). Groups are illustrated with different colours (green = infants at typical likelihood of ASD or ADHD; violet = infants at elevated likelihood of ASD; grey = infants at elevated likelihood of ADHD; orange = infants at elevated likelihood of ASD and ADHD). Notes: (1) Fit lines are presented for an average of all infants. (2) The participant with a TSI < − 1 in **a** does not appear in **b** and **c** since this infant did not contribute ECBQ data

The hierarchical linear regression with TSI as predictor and ADOS-S CSS (log) as outcome was statistically significant, F(1,77) = 15.795, *p* < .001, *R*^2^_adj_ = .159, indicating that infants with lower neural repetition suppression of tactile stimulation at 10 months exhibited higher levels of ASD traits at 24 months. In step 2, the likelihood factors and the interaction terms were entered as predictors (ASD-L, ADHD-L, interaction between ASD-L and TSI, interaction between ADHD-L and TSI). The model remained statistically significant, F(5,73) = 4.13, *p* = .002, *R*^2^_adj_ = .167, but did not account for a significantly higher proportion of variance relative to a model with only TSI as predictor, F change (4,73) = 1.17, *p =* .329. There was no evidence of moderation by either ASD likelihood (*β* = .059, *p* = .852) or ADHD likelihood (*β* = .003, *p* = .987). The results from step 2 did not change when ECBQ activity was partialled out, F(6,62) = 4.087, *p* = . 002, *R*^2^_adj_ = .214, 95% CI for B [− .170, .163]; when ECBQ inhibitory control was partialled out, F(6,61) = 4.226, *p* = . 001, *R*^2^_adj_ = .294, 95% CI for B [− .189, .071], see Fig. 5a and Table 3.

**Table 3:** Correlation coefficients (Pearson *r*) for associations between S1 α desynchronization, S2-S1 α desynchronization (10 months) and ADOS-2 CSS (log) (24 months) or ECBQ activity (24 months) or ECBQ inhibitory control in the entire sample

Entire sample	ADOS-2 CSS (log)	ECBQ activity	ECBQ inhibitory control
α S1	− .064	− .107	.116
α S2–S1	− .413**	− .165	.110

**p < .001

#### Associations with ADHD traits at 24 months

The hierarchical linear regression with S1 alpha amplitude desynchronization as predictor and ECBQ activity as outcome was not statistically significant, F(1,69) = .797, *p* = .375, *R*^2^_adj_ = .011, and that with ECBQ inhibitory control as outcome was also not statistically significant, F(1,68) = .920, *p* = .341, *R*^2^_adj_ = .013. Both results did not change when ADOS-2 CSS was partialled out: for ECBQ activity, F(2,66) = .497, *p* = .611, *R*^2^_adj_ = .000, 95% CI for B [− .225, .371] and for ECBQ inhibitory control, F(2,65) = 1.716, *p* = .188, *R*^2^_adj_ = .021, 95% CI for B [− .742, .071].

The hierarchical linear regression with TSI as predictor and ECBQ activity as outcome and was not statistically significant, F(1,69) = 1.92, *p* = .170, *R*^2^_adj_ = .013, and that with ECBQ inhibitory control as outcome was also not statistically significant, F(1,68) = .838, *p* = .363, *R*^2^_adj_ = .012. Both results did not change when ADOS-2 CSS was partialled out: for ECBQ activity, F(2,66) = .947, *p* = .393, *R*^2^_adj_ = .000, 95% CI for B [− .358, .297] and for ECBQ inhibitory control, F(2,65) = 1.329, *p* = .272, *R*^2^_adj_ = .010, 95% CI for B [− .754, .174], see Fig. 5b and c.

### Mediating/moderating effect of tactile sensory seeking

Results from previous analyses indicated that reduced neural repetition suppression of tactile stimulation (TSI) is a marker significantly capturing the effect of ASD likelihood status at 10 months and predicting ASD traits at 24 months.

We then assessed whether tactile sensory seeking significantly mediated or moderated the relationship between TSI and later ASD traits (see SM3, Fig. 3). To conclude that tactile sensory seeking mediates the relationship between early neural repetition suppression of tactile stimulation and later ASD traits, a significant *indirect effect* of neural repetition suppression on ASD traits, through tactile sensory seeking, should be observed. Two pathways comprise the indirect effect: (1) “a path” represents the relation between neural repetition suppression and tactile sensory seeking and (2) “b path” represents the relation between neural repetition suppression and ASD traits, controlling for tactile sensory seeking. An indirect effect is statistically significant when the confidence interval for the product of the unstandardized coefficients for these two paths does not include zero.

To conclude that tactile sensory seeking moderates the relationship between early neural repetition suppression of tactile stimulation and later ASD traits, a significant *interaction effect* between neural repetition suppression and tactile sensory seeking on ASD traits should be observed.

In the following mediation and moderation analyses, bias-corrected confidence intervals for effects of interest were generated using 5000 bootstrap samples with the confidence level set at 95%.

#### Mediation model

The direct effect of TSI on ADOS-2 CSS (log) was statistically significant at 95% CI [− 1.759, − .537]. The direct effect of tactile sensory seeking on ADOS-2 CSS (log) was also statistically significant at 95% CI [.091, .547]. No evidence for an indirect effect of TSI on ADOS-2 CSS (log) through tactile sensory seeking emerged: (1) “a path” from tactile sensory seeking to TSI was not statistically significant at 95% CI [− 1.066, .226] and (2) “b path” from TSI to ADOS-2 CSS (log) controlling for tactile sensory seeking was not statistically significant at 95% CI [− .408, .001].

#### Moderation model

**Fig. 6:**
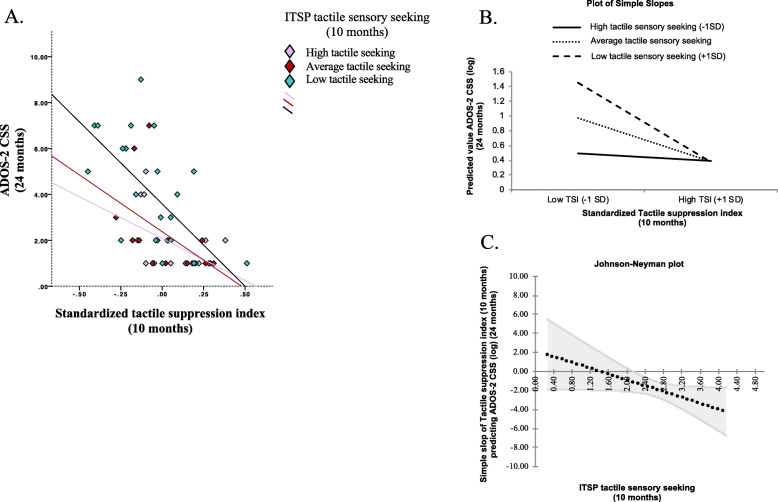
**a** Scatterplot illustrating the moderating effect of tactile sensory seeking (10 months) on the association between tactile suppression index (S2–S1 α amplitude) at 10 months and ADOS-2 CSS at 24 months. **b** Plot of simple slopes illustrating the interaction effect of tactile sensory seeking: the association between tactile suppression index and ADOS-2 CSS (log) is significant for average and low tactile sensory seeking (*p* < .001) but not significant for high tactile sensory seeking (*p* = .688). **c** Johnson-Neyman plot illustrating the region of significance of the moderator: the association between tactile suppression index and ADOS-2 CSS (log) is not significant for values of tactile sensory seeking ≤ 2.13 (i.e. high tactile seeking)

The interaction effect between TSI and tactile sensory seeking on ADOS-2 CSS (log) was statistically significant at 95% CI [− 2.919, − .154], indicating a moderation role of tactile sensory seeking. Analysis of the conditional effects (i.e. spotlight analysis) indicated that TSI significantly predicted ADOS-2 CSS when tactile sensory seeking was low (95% CI [− 3.340, − 1.086], *p* < .001) or average (95% CI [− 1.807, − .614], *p* < .001) but not high (95% CI [− 1.241, .825], *p* = .688). Johnson-Neyman analysis (i.e. floodlight analysis) indicated that the association between tactile suppression index and ADOS-2 CSS (log) was not significant for values of tactile sensory seeking ≤ 2.13 (i.e. high tactile seeking), see Fig. 6 and Table 4.

**Table 4:** Conditional effects of tactile suppression index (10 months) on ADOS-2 CSS (24 months) depending on ITSP tactile sensory seeking (10 months)

ITSP tactile sensory seeking	B	*p*	95% CI
Mean +1SD (low seeking)	− 2.213**	< .001	− 3.340, − 1.086
At the mean (average seeking)	− 1.210**	< .001	− 1.807, − .614
Mean −1SD (high seeking)	− 0.208	.688	− 1.241, .825

**p < .001

### Additional analyses

Tactile sensory avoiding may be seen as the opposite to tactile sensory seeking and may better capture its mediating function. We found no evidence of tactile sensory avoiding (from ITSP at 10 months) mediating or moderating the relationship between TSI at 10 months and ADOS-2 CSS at 24 months (see SM2).

## Discussion

The goal of the present study was to investigate behavioural and neural markers of tactile sensory processing in 10-month-old infants at elevated likelihood of ASD or ADHD and infants at typical likelihood of the disorders. First, we quantified infants’ behavioural responses to repeated tactile stimulation, as objective assessment of infants’ behavioural sensitivity. We observed that all infants, independent of their likelihood status, exhibited a decrease in screen-directed looking and an increase in body movement from the pre to the post-stimulus phase. Previous reports of behavioural sensitivity used parental reports [7, 30, 97]. Other laboratory-based experimental and observational measures failed to report differences in behavioural sensitivity. For example, behavioural sensitivity measured during a structured observational task comprising self-directed and examiner-directed tactile stimulation (i.e. Tactile Defensiveness and Discrimination Test Revised; TDDT-R) did not associate with ASD core symptoms, as measured by the ADOS and ADI-R [28]. Further, there is evidence that parental reports do not always correlate with clinical or experimental observations [28, 61]. In our study, a parent-reported measure (i.e. behavioural sensitivity to tactile stimulation quantified through the sensory sensitivity and low registration quadrants of the ITSP) was also unaffected by likelihood status (see SM2 for this analysis). Thus, this evidence suggests that the absence of behavioural differences at 10 months may not be a consequence of the coding approach used. Nonetheless, it is important to note that the coding approach adopted was not designed to detect fine-grained differences in behavioural sensitivity. Thus, we cannot rule out the possibility that subtle differences in behavioural manifestations existed between the groups. Alternatively, it is also possible that stronger or more aversive stimulation may be needed to observe an effect of likelihood status on behavioural sensitivity to tactile input. While no significant results emerged from the investigation of the effect of likelihood status on behavioural or parent-reported markers of sensitivity to tactile stimulation, we did observe significant associations between change in movement from the pre-stimulus to the post-stimulus phase and parental reports of behavioural sensitivity to tactile stimulation (as assessed through the ITSP [24]; see SM2), thus suggesting that both experimental observation and parental reports were capturing meaningful variation in infants’ sensitivity to tactile input. In particular, results indicated that infants manifesting elevated increase in movement from the pre-stimulus to the post-stimulus phase were reported by parents to display enhanced tactile sensory sensitivity (and enhanced tactile low registration, see SM2 for further discussion of this result). Altogether, this evidence highlights the importance of an integrated approach, combining experimental and parent-reported measures, to investigate tactile sensory processing in early development.

In contrast to our hypothesis, response strength to the first stimulus in the pair did not associate with participants’ likelihood status. Based on previous studies, we predicted neural hyposensitivity to S1 to associate with an ADHD likelihood status and to predict later ADHD traits [23, 52, 78, 79]. Neither neural sensitivity to S1 differentiated infants with an ADHD likelihood status nor did it predict later activity or inhibitory control traits measured with the parent-reported ECBQ. The lack of association between neural sensitivity to S1 and the ADHD likelihood status or later ADHD traits is surprising, given that theoretical accounts often assume the hyperactivity and reduced inhibitory control characteristic of ADHD to compensate for sensory hyposensitivity (e.g. [101]). Experimental evidence in support of this hypothesis remains scarce. For example, Bijlenga et al. [12] failed to document hyposensitive-related behaviours in adults with ADHD. We need to note that, in our study, we used the parental report ECBQ to quantify ADHD traits in 24-month-old toddlers. Although ECBQ activity and inhibitory control at 24 months associate with ADHD symptoms at 7 years [90], these measures may not capture the whole spectrum of later ADHD symptomatology.

Neural sensitivity to S1 also did not associate with the ASD likelihood status or predict later ASD traits, quantified through ADOS-2 CSS. Although one report documented a significant positive association between neural sensitivity to S1 and ASD traits in 8–18-year-old participants with ASD [51], the majority of animal and human research converges in suggesting that reduced neural repetition suppression of tactile stimulation characterizes this condition [36, 40, 78–80]. In other sensory modalities (i.e. auditory), reduced neural repetition suppression in the absence of neural hypersensitivity in ASD has also been documented [65]. Reduced neural repetition suppression, rather than increased response to a single stimulus, may account for the behavioural profile of sensory hypersensitivity documented in the early development of ASD [7, 16, 97].

In addition to assessing infants’ neural sensitivity to S1, our task was designed to measure neural repetition suppression of tactile stimulation (S2–S1). Atypicalities in neural repetition suppression have been documented in populations with ASD and ADHD, with accumulating evidence coming from the auditory modality [65, 70, 92], including in populations of infants at elevated likelihood of ASD [37, 53, 89]. Hence, we predicted to observe significant effects of ASD and ADHD likelihood status on neural repetition suppression. While significant reduction in alpha amplitude desynchronization to repeated tactile stimulation only occurred in infants at typical likelihood of the conditions, only the ASD likelihood status impacted as a factor on this measure. This result was reinforced by the finding of a specific association between neural repetition suppression of tactile stimulation at 10 months and ASD traits at 24 months, across the entire sample. This association was not moderated by likelihood status, suggesting that the pathway identified is independent of familial contributions. Previous work questioned whether ASD manifests the same phenotype when accompanied by ADHD [90, 98]. Our results suggest that a common pathway to later ASD traits exists in infants at elevated likelihood of ASD or ADHD. However, as discussed in SM1 (“Clinical assessment”), it remains likely that within families with ASD, rates of actual ADHD were higher than those captured by our 1/0 diagnostically-based rating system, reflecting the fact that in the UK clinically diagnosed prevalence rates of ADHD are lower than population prevalence estimates (which is not the case for ASD; see [85]). While our screening method was designed to reduce group mischaracterization (given that screening occurred for those families who reported ADHD concerns), it remains possible that some infants were mischaracterized into the EL-ASD group when they should have been in the EL-ASD+ADHD group. In turn, this mischaracterization may have driven the lack of a moderating effect of likelihood status on the association between neural repetition suppression of tactile stimulation at 10 months and ASD traits at 24 months.

Alteration in the excitation/inhibition balance of neural connectivity has been proposed as a mechanism underlying many of the manifestations occurring in ASD and ADHD, including atypical repetition suppression [50, 55, 81]. Since repetition suppression partly reflects GABAergic inhibition of glutaminergic pyramidal cells in the interneuronal network [54, 55], reduced inhibition in the somatosensory cortex could underlie the atypical repetition suppression documented in our study. Additional perceptual phenomena that have been linked to alteration in the excitation/inhibition balance include binocular rivalry, spatial suppression/gain control and orientation discrimination (for reviews see, [22, 82]). Thus, extending to the tactile modality evidence of atypical neural repetition suppression, our findings suggest that such atypicality may be domain-general rather than tied to a specific sensory modality. Gathering evidence of atypical neural repetition suppression in the tactile modality is essential, given that touch is the first sense to develop and the mean through which infants learn about the environment and themselves [13]. Further, touch contributes to the development of early social bonds [15, 26, 58]. Indeed, it has been proposed that early tactile dysfunction may exacerbate later ASD symptomatology by triggering compensatory strategies aimed at minimizing tactile input [62].

Thus, as a final step in our analytical pipeline, we sought to explore the effect of tactile sensory seeking as a potential mediator or moderator of the relationship between early atypical neural response and later ASD traits. Decreased sensory seeking is often reported in infants later developing ASD [10, 67, 96] and may represent a compensatory strategy adopted by infants to minimize sensory input [45, 46, 67]. However, reduced sensory seeking could also limit infants’ opportunities for learning and socialization, thus exacerbating later ASD traits. Contrary to this hypothesis, we found no evidence of a mediating role of tactile sensory seeking at 10 months. In contrast, we found that tactile sensory seeking significantly moderated the association between 10-month tactile neural repetition suppression and 24-month ADOS-2 CSS. This moderation effect was specific to seeking and did not extend to other sensory behaviours like avoiding (see SM2). Thus, at the same level of neural repetition suppression of tactile stimulation, infants reported by parents as concurrently seeking more tactile input developed lower ASD traits at 24 months. Thus, tactile sensory seeking could represent an independent compounding factor, moderating the association between early reduced neural repetition suppression and later ASD traits. Indeed, previous research suggests that tactile sensory seeking does not always associate with elevated sensory responsiveness [10, 75]. We speculate that reduced neural repetition suppression may interfere with learning by slowing prior updating [74] (see also associations with Mullen Scales in SM2). From this perspective, increased tactile sensory seeking may have a protective role during development by widening opportunities for learning and socialization.

## Conclusions

Overall, our study presents the first evidence of atypical neural repetition suppression of tactile stimulation in infants at elevated likelihood of ASD. We demonstrate that reduced tactile neural repetition suppression is an early marker of later ASD traits in infants at elevated likelihood of ASD or ADHD, suggesting that a common pathway to later ASD exists across these different familial backgrounds. Further, we establish tactile sensory seeking as a moderator of the association between early reduced neural repetition suppression and later ASD traits (i.e. high tactile seeking mitigates the association between early reduced neural repetition suppression and later ASD traits). Thus, we identify a pathway to the emergence of ASD traits and emphasize the need to discover additional factors for the development of ADHD traits. Future research should assess whether continuity exists between the marker identified in the current study and the heterogeneous spectrum of sensory features documented later in development, including sensory hyper/hyposensitivity manifestations.

## Supplementary Information

**Additional file 1:**

## Data Availability

The data is available from the BASIS network through a set of sharing procedures that comply with the ethical permissions under which this highly sensitive dataset was collected (see www.basisnetwork.org).

## Abbreviations

ADHD: Attention deficit hyperactivity disorder
ADI-R: Autism Diagnostic Interview-Revised
ADOS-2: Autism Diagnostic Observation Schedule, Second edition
ADOS-2 CSS: ADOS-2 Calibrated Severity Scores
ASD: Autism spectrum disorder
BOLD: Blood-oxygen-level-dependent
CAARS: Conners Adults ADHD Rating Scale
CI: Confidence interval
CH: Electrode channel
DSM-5: Diagnostic and Statistical Manual of Mental Disorders, 5^th^ edition
DSM-IV: Diagnostic and Statistical Manual of Mental Disorders, 4^th^ edition
ECBQ: Early Childhood Behaviour Questionnaire
EEG: Electroencephalography
EL-ADHD: Elevated likelihood of attention deficit hyperactivity disorder
EL-ASD: Elevated likelihood of autism spectrum disorder
EL-ASD+ADHD: Elevated likelihood of autism spectrum disorder and attention deficit hyperactivity disorder
GABA: Gamma-aminobutyric acid
ICC: Intra-class correlation coefficient
ICD-10: International Statistical Classification of Diseases and Related Health Problems, 10^th^ revision
ISI: Inter-stimulus interval
ITSP: Infant-Toddler Sensory Profile
MSEL EL: Mullen Scales for Early Learning Expressive Language
MSEL ELC: Mullen Scales for Early Learning Early Composite Score
MSEL FM: Mullen Scales for Early Learning Fine Motor Score
MSEL GM: Mullen Scales for Early Learning Gross Motor Score
MSEL RL: Mullen Scales for Early Learning Receptive Language Score
MSEL VR: Mullen Scales for Early Learning Visual Reception Score
Q-CHAT: Quantitative CHecklist for Autism in Toddlers
S1: First vibrotactile stimulus
S2: Second vibrotactile stimulus
SPS: Sensory Processing Scale
TDDT-R: Tactile Defensiveness and Discrimination Test Revised
TL: Typical likelihood of autism spectrum disorder and attention deficit hyperactivity disorder
TSI: Tactile Suppression Index
