# Supplementary material for: Behavioural and neural markers of tactile sensory processing in infants at elevated likelihood of autism spectrum disorder and/or attention deficit hyperactivity disorder
Source: J Neurodev Disord. 2021 Jan 4;13:1. doi: 10.1186/s11689-020-09334-1 (PMC7780639; doi:10.1186/s11689-020-09334-1)
Supplement: Supplementary file 1 — Additional file 1: [file 11689_2020_9334_MOESM1_ESM.pdf]

Supplementary materials

Behavioural and neural markers of tactile sensory processing in infants at elevated likelihood of Autism Spectrum Disorder and/or Attention Deficit Hyperactivity Disorder

Elena Serena Piccardi[1], Jannath Begum Ali[1], Emily J.H. Jones[1], Luke Mason[1], Tony Charman[2], Mark H. Johnson[1,3], Teodora Gliga[1,4] & BASIS/STAARS Team[ϕ]

[1] Centre for Brain and Cognitive Development, Department of Psychological Sciences, Birkbeck, University of London (UK)

[2] Institute of Psychiatry, Psychology & Neuroscience, Psychology Department, King's College London (UK)

[3] Department of Psychology, Cambridge University, Cambridge (UK)

[4] Department of Psychology, University of East Anglia, Norwich (UK)

ϕ BASIS/STAARS Team members listed in the Acknowledgements

Corresponding author: Elena Serena Piccardi

Address: Centre for Brain and Cognitive Development, Malet Street, WC1E 7HX, London (UK)

E-mail address: epicca01@mail.bbk.ac.uk

Telephone number: 0207 079 0896

Conflict of interest statement

The authors declare that they have no conflict of interest.

SM1- Methods

Clinical assessment

Information about diagnostic status was ascertained through a number of methods. Before families enrolled in the study, a telephone screening form was used to determine the presence of ASD and ADHD in family members. During their infant's visit to the lab, the parent/caregiver also completed a "Medical and Psychiatric History Interview" (Appendix A) with the researcher. The telephone screening form and this formal interview at study visit were the primary sources of information about diagnostic status. In addition, we asked for medical updates at each study visit and re-administered the Medical and Psychiatric History Interview at the 3-year timepoint. We also requested diagnostic letters and asked parents to complete the DAWBA ASD and ADHD sections (Goodman et al., 2000) and these were reviewed by the senior clinician (TC). In addition, parents completed the Conners (Conners, 2008) (for ADHD) and the Social Communication Questionnaire (Rutter, Bailey & Lord, 2003) and Social Responsiveness Scale (Constantino, 2002; for ASD) on the family member with a diagnosis and, where possible, all other family members. This information was used to characterise our sample rather than for exclusionary purposes since, in the UK, NHS clinical diagnoses follow a gold-standard procedure including collation of information from parents, teachers and from in-person assessment that is beyond the scope of this study and more accurate than questionnaire measures.

Up to 30% of children with ASD meet criteria for ADHD when prospectively assessed (Simonoff et al., 2008). In clinical practice, the prevalence of dual diagnosis is lower (Russell, Rodgers, Ukoumunne & Ford, 2014). Given the nature of the co-occurrence between ASD and ADHD and our longitudinal study, sometimes family members would have a suspected diagnosis of ADHD at study entry that would be

confirmed later in the study. On other occasions, a family would enroll on the basis of an ASD diagnosis in an older sibling but by the end of the study, they would report that the same sibling was now undergoing assessment for suspected additional ADHD. Where possible, families who reported suspected ADHD at study entry were screened using a shortened version of the Conners. Families who were screened positive on this instrument were then included as a confirmed case. However, it remains likely that within families with ASD, rates of actual ADHD are higher than those captured by our 1/0 diagnostically-based rating system. Families where there was significant diagnostic uncertainty about the presence of either ASD or ADHD were removed in a sensitivity analysis to check whether results differed substantially (n=7 cases in the entire sample; of these n=2 participants contributed EEG and behavioural data and n=0 participants contributed outcome data). Results of this re-analysis are reported in SM2 section: Sensitivity analysis.

ITSP list of items and reliability assessment

Four items contributed to the ITSP tactile sensory seeking score. These items ask parents to rate on a 5-point scale (1 = almost always; 5 = almost never) if the child enjoys playing with food (item 31); if the child seeks opportunities to feel vibrations (for example, stereo speakers, washer, dryer) (item 32); if the child enjoys splashing during bath time (item 34); if the child uses hands to explore food and other textures (item 35). We investigated reliability of the ITSP sensory seeking items in the tactile modality for participants contributing to the EEG analyses by extracting Cronbach's α and composite reliability (CR). CR was extracted since Cronbach's α depends on the number of items and tends to underestimate internal consistency with fewer items

(Tavakol & Dennick, 2011). At 10 months, Cronbach's α = 0.527 and CR = 0.758, indicating satisfactory internal consistency.

SM2 - Results

Sensitivity analysis

All the analyses reported in the manuscript were re-run after removing n=2 cases contributing behavioural and EEG data for whom significant diagnostic uncertainty existed.

Behavioral results (i.e. looking and body movement to tactile stimulation) did not differ from those reported in the manuscript. Neural results were also replicated following removal of uncertain cases. Precisely, the significant effect of the ASD likelihood status on TSI was replicated, $F(1,85) = 5.30$, $p = .024$, $\eta^2 = .059$. The non-significant results from the investigation of infants' neural sensitivity (S1) and neural repetition suppression of tactile stimulation (TSI) were replicated. The longitudinal associations between EEG measures at 10 months and ASD or ADHD traits at 24 months remained unchanged since none of the removed cases contributed outcome data. For the same reason, results from the analyses investigating the potential mediating/moderating effect of tactile sensory seeking remained unaltered.

Effect of likelihood status on behavioural markers of tactile sensitivity in the extended sample

It could be argued that infants excluded from the EEG analyses due to fussiness/movement artifacts may be the most hypersensitive to tactile stimulation. Therefore, we re-ran the analyses on behavioural markers of sensitivity to tactile stimulation assessed during the experiment following inclusion of those infants excluded from the EEG analyses due to fussiness/excessive movement artifacts. 34

out of 38 infants excluded from the EEG analyses had usable behavioural data. The remaining 4 infants completed less than 3 experimental trials and, thus, were not considered for behavioural coding.

For screen-directed looking, a main effect of stimulation (pre *vs*. post-stimulus phase) emerged, $F(1,120) = 25.46$, $p < .001$, $\eta^2 = .175$, indicating looking away from the screen after receiving the tactile stimulation. There was no significant interaction between stimulation and ASD likelihood status, $F(1,120) = 0.407$, $p = .525$, $\eta^2 = .003$, or between stimulation and ADHD likelihood status, $F(1,120) = 1.63$, $p = .203$, $\eta^2 = .013$. There was also no significant three-way interaction between stimulation, ASD and ADHD likelihood status, $F(1,120) = 1.31$, $p = .254$, $\eta^2 = .011$.

For body movement, a main effect of stimulation (pre *vs*. post-stimulus phase) emerged, $F(1,120) = 47.89$, $p < .001$, $\eta^2 = .285$, indicating increased movement after receiving the tactile stimulation. There was, however, no significant interaction between stimulation and ASD likelihood status, $F(1,120) = .498$, $p = .482$, $\eta^2 = .004$. The interaction between stimulation and ADHD likelihood status reached statistical significance, $F(1,120) = 6.28$, $p = .014$, $\eta^2 = .050$. However, post-hoc pairwise tests did not survive correction for multiple comparisons (Bonferroni correction; all p_s >.05). There was no significant three-way interaction between stimulation, ASD and ADHD likelihood status, $F(1,120) = .612$, $p =.436$, $\eta^2 = .005$.

Overall, these results replicate in a larger sample of infants the evidence reported in the manuscript and confirm that infants' likelihood status did not significantly differentiate behavioural markers of sensitivity to tactile stimulation during the task.

Parental reports of infants' behavioural sensitivity to tactile stimulation

A set of analyses was performed to assess whether infants' likelihood status differentiated parental reports of behavioural sensitivity to tactile stimulation (i.e. quantified through 10-month ITSP; Dunn, 2002).

ITSP sensory sensitivity and low registration scores for the tactile modality were computed for each 10-month-old infants contributing EEG data. The tactile low registration variable significantly violated normality assumptions (Shapiro-Wilk, $p < .001$; Skewness = -.720, SE = .274; Kurtosis = .257, SE = .541) and was log transformed prior to the analyses. The univariate ANOVA on tactile sensory sensitivity indicated no significant main effect of ASD, $F(1,71) = .742$, $p = .392$, $\eta^2 = .010$, or ADHD, $F(1,71) = .061$, $p = .805$, $\eta^2 = .001$. Further, no significant interaction between ASD and ADHD likelihood status emerged, $F(1,71) = .267$, $p = .607$, $\eta^2 = .004$. The univariate ANOVA on tactile low registration (log) indicated no significant main effect of ASD, $F(1,71) = .087$, $p = .769$, $\eta^2 = .001$. The main effect of ADHD failed to reach statistical significance, $F(1,71) = 3.77$, $p = .056$, $\eta^2 = .050$. No significant interaction between ASD and ADHD emerged, $F(1,71) = 0.64$, $p = .800$, $\eta^2 = .001$.

We also re-ran the analyses on the extended sample of 10-month-old infants, which included participants who contributed EEG data and participants excluded from the EEG analyses due to fussiness/movement artifacts. The univariate ANOVA on tactile sensory sensitivity indicated no significant main effect of ASD, $F(1,111) = .353$, $p = .554$, $\eta^2 = .003$, or ADHD, $F(1,111) = .554$, $p = .458$, $\eta^2 = .005$. Further, no significant interaction between ASD and ADHD likelihood status emerged, $F(1,111) = .461$, $p = .499$, $\eta^2 = .004$. The univariate ANOVA on tactile low registration (log) indicated no significant main effect of ASD, $F(1,111) = .087$, $p = .769$, $\eta^2 = .001$, or

ADHD, F(1,111)= 2.43, p = .122, η^2 = .021. No significant interaction between ASD and ADHD emerged, F(1,111) = 0.60, p = .807, η^2 = .001.

Overall, results from these analyses indicate that likelihood status was not differentiating parental reports of infants' behavioural sensitivity to tactile stimulation. This evidence aligns to results reported in the manuscript, suggesting that the absence of behavioural differences at 10 months may not be a consequence of the coding approach used.

Associations between behavioural markers of sensitivity to tactile stimulation assessed during the EEG task and assessed through parental reports

To investigate the consistency between behavioural markers of sensitivity to tactile stimulation (assessed during the experiment) and parental reports of sensitivity to tactile stimulation (assessed through the ITSP sensory sensitivity and low registration quadrants in the tactile modality), a set of Pearson correlations was run. These analyses were conducted on the extended participant sample (which included infants who contributed to the EEG analyses and infants excluded from the EEG analyses due to fussiness/excessive movement artifacts).

There was no significant association between differential looking score (post S2 - pre S1) and ITSP tactile sensory sensitivity, R (98) = -.028, p = .728, R^2 =.001; and ITSP tactile low registration (log), R (98) = -.119, p = .239, R^2 =.014. Contrarily, significant associations emerged between differential moving score (post S2- pre S1) and ITSP tactile sensory sensitivity, R (98) = -.233, p = .021, R^2 =.054; and ITSP tactile low registration (log), R (98) = -.223, p = .026, R^2 =.050. These results indicated that infants manifesting elevated increase in movement following the tactile stimulus were rated by parents as displaying enhanced sensory sensitivity and enhanced low

registration in the tactile modality. The direction of the association between change in movement during the task and tactile low registration is surprising, given that the low registration quadrant of the ITSP should capture manifestations consistent with behavioural hyposensitivity (whereas the sensory sensitivity quadrant should capture manifestations consistent with behavioural hypersensitivity). However, in our sample, the tactile low registration quadrant positively associated with the tactile sensory sensitivity quadrant, R (107) = .429, p < .001, R^2 =.184, suggesting that the two ITSP quadrants were not capturing opposite constructs. We exclude poor internal consistency of the overall low registration quadrant as an explanation for this result, (Cronbach's α = 0.824), as well as low convergent validity between the item contributing to the tactile low registration quadrant and items contributing to the overall low registration quadrant (correlation coefficients ranging from R=.150 to R=.496, with the exception of the correlation with the item constituting the low registration quadrant in the vestibular modality, R = -.016). Conversely, we hypothesize that the format of the question designed to capture tactile low registration was not ideal to quantify manifestations consistent with behavioural hyposensitivity to tactile input in 10-month-old infants (i.e. "My child bumps into things, seeming to not notice objects in the way").

Associations between neural markers and parent-reported ASD traits

Associations with ASD traits at 24 months. Q-CHAT scores significantly violated normality assumptions (Shapiro-Wilk, p < .001; Skewness = .943, SE = .226; Kurtosis = 1.164, SE = .447). Log transformation did not improve the data distribution (Shapiro-Wilk, p = .001; Skewness = -.778, SE = .226; Kurtosis = 2.485, SE = .447). Thus, Spearman correlations were run to assess the associations between neural markers and this outcome measure.

The Spearman correlation between Q-CHAT and S1 alpha desynchronization was not statistically significant, Rho (72) = -.054, *p* = .322; and TSI was also not statistically significant, Rho (72) = -.115, *p* = .165. Given that TSI at 10 months significantly predicted ADOS-2 CSS at 24 months, we also assessed the concordance between Q-CHAT and ADOS-2 CSS. There was low concordance between the measures, Rho (70) = .244, *p* = .039.

Overall, these results indicate that parent-reported ASD traits (quantified through Q-CHAT at 24 months) did not associate with neural sensitivity to and suppression of repeated tactile stimulation. Tactile neural repetition suppression at 10 months significantly predicted later ASD traits as measured by the researcher-rated observational measure ADOS-2 CSS at 24 months. Indeed, in the current study, concordance was low between ADOS-2 CSS and Q-CHAT. Low-to-moderate correlation between observational and parent report measures is commonly reported in older samples of children with ASD (e.g. Charman et al., 2007; Evers et al., 2020; Lord et al., 2006) and between clinician ratings and parent ratings of ADHD (Nobel et al., 2019), which is why best practice in diagnostic clinical assessments is to use both methods (Lord et al., 2020). In younger toddlers, concordance between parental reports and standardised clinical assessments of ASD or ADHD traits may be even lower since atypical manifestations are less prominent. Another possibility is that Q-CHAT and the neural measures quantified in the present study captured different constructs. Thus, both the significant associations between neural markers of tactile sensory processing and later ASD traits (quantified through clinician observation) and the non-significant associations between the same neural markers and later ADHD traits (quantified through parental report) in the manuscript must be followed-up using both observational and parent report assessments of ASD and ADHD traits at 3 years.

Associations between behavioural markers and later ASD or ADHD traits

Associations with ASD traits at 24 months. The hierarchical linear regression with differential looking score (post S2 - pre S1) as predictor and ADOS-2 CSS (log) as outcome was not statistically significant, F(1,76) = .013, p = .909, R^2_{adj} = .000. The result did not change when ECBQ activity was partialled out, F(2,65) = .154, p = .857, R^2_{adj} = .000, 95% CI for B, [-.139, .244]; when ECBQ inhibitory control was partialled out, F(2,64) = 2.216, p = .117, R^2_{adj} = .036, 95% CI for B, [-.283, -.007].

The hierarchical linear regression with differential moving score (post S2 - pre S1) as predictor and ADOS-2 CSS (log) as outcome was not statistically significant, F(1,76) = 2.628, p = .109, R^2_{adj} = .021. The result trended towards significance when ECBQ activity was partialled out, F(2,65) = 3.112, p = .051, R^2_{adj} = .059, 95% CI for B, [-.192, .186]; reached statistical significance when ECBQ inhibitory control was partialled out, F(2,64) = 4.883, p = .011, R^2_{adj} = .105, 95% CI for B, [-.256, .012].

Overall, these results indicate that infants' relative looking following the tactile stimulation at 10 months did not significantly predict ASD traits at 24 months. Infants' relative movement following the tactile stimulation at 10 months significantly predicted ASD traits at 24 months, with infants manifesting more movement reporting fewer ASD-related traits, but this association was only apparent when controlling for ADHD traits.

Associations with ADHD traits at 24 months. The hierarchical linear regression with differential looking score (post S2 - pre S1) as predictor and ECBQ activity as outcome was not statistically significant, F(1,68) = .016, p = .900, R^2_{adj} = .000; and ECBQ inhibitory control as outcome was also not statistically significant, F(1,67) = .358, p = .552, R^2_{adj} = .000. Both results did not change when ADOS-2 CSS (log) was partialled out: for ECBQ activity, F(2,65) = .155, p = .856, R^2_{adj} = .000, 95%

CI for B, [-.231, .407]; for ECBQ inhibitory control, F(2,64) = 2.425, p = .097, R^2_{adj} = .041, 95% CI for B, [-.870, -.022].

The hierarchical linear regression with differential moving score (pre S2 – pre S1) as predictor and ECBQ activity as outcome trended towards statistical significance, F(1,68) = 3.908, p = .052, R^2_{adj} = .040; with ECBQ inhibitory control as outcome was not statistically significant, F(1,67) = 1.347, p = .250, R^2_{adj} = .005. Both results did not change when ADOS-2 CSS (log) was partialled out: for ECBQ activity, F(2,65) = 2.004, p = .143, R^2_{adj} = .029, 95% CI for B, [-.329, .320]; for ECBQ inhibitory control, F(2,64) = 2.446, p = .095, R^2_{adj} = .042, 95% CI for B, [-.847, .039].

Overall, these results indicate that neither infants' relative looking nor infants' relative movement following the tactile stimulation at 10 months significantly predicted ADHD traits at 24 months.

Effect of likelihood status on tactile sensory seeking

We investigated the effect of likelihood status on the ITSP tactile sensory seeking scores measured at 10 months for infants contributing to the EEG analysis. A univariate ANOVA with tactile sensory seeking as dependent variable, ASD and ADHD likelihood status (dummy coded) as factors was run. The analysis revealed a significant main effect of ASD, F(1,75) = 10.53, p = .002, η^2 = .123. No significant main effect of ADHD was observed, F(1,75) = .002, p = .964, η^2 = .00. Further, there was no significant interaction between ASD and ADHD, F(1,75) = .292, p = .590, η^2 = .004. See SM3 Figure 2.

Overall, results from these analyses indicate that the ASD likelihood significantly impacted as a factor on infants' tactile sensory seeking, with infants having an elevated likelihood of ASD reporting significantly lower tactile sensory seeking than infants without an ASD likelihood.

Mediating/moderating effect of tactile sensory avoiding

ITSP sensory avoiding scores for the tactile modality were computed for each 10-month-old infants. First, descriptive investigation of the variable distribution indicated that 83.2% of the data fell within an interval ranging from 3.5 to 5 (i.e. indexing low tactile sensory avoiding on the ITSP), thus suggesting that infants as young as 10 months may not yet possess a sufficient skills repertoire to display active avoidance behaviours.

Since tactile sensory avoiding could be opposite to tactile sensory seeking, we assessed the relationship between the two ITSP measures. The Pearson correlation between the measures was not statistically significant, r (73) = .014, p = .905, disconfirming the potential link between the two quadrants within the tactile domain of the ITSP. We subsequently assessed the explanatory power of tactile sensory avoiding as a mediator or moderator of the relationship between 10-month neural repetition suppression of tactile stimulation and 24-month ASD traits.

Mediation model. The direct effect of TSI on ADOS-2 CSS (log) was statistically significant at 95% CI, [-1.759, -.537]. The direct effect of tactile sensory avoiding on ADOS-2 CSS (log) was not statistically significant at 95% CI, [-.260, .276]. No evidence for an indirect effect of TSI on ADOS-2 CSS (log) through tactile sensory avoiding emerged: 1] “a path” from tactile sensory avoiding to TSI was not statistically significant at 95% CI, [-.912, .444]; 2] “b path” from TSI to ADOS-2 CSS (log) controlling for tactile sensory seeking was not statistically significant at 95% CI, [-.112, .087].

Moderation model. The interaction effect between TSI and tactile sensory avoiding on ADOS-2 CSS (log) was not statistically significant at 95% CI, [-2.968, .004], disconfirming the moderation role of tactile sensory avoiding.

Overall, these results disconfirm the existence of a link between parental reports of tactile sensory seeking and avoiding behaviours at 10 months. Further, no evidence of tactile sensory avoiding mediating or moderating the association between neural repetition suppression of tactile stimulation at 10 months and ASD traits at 24 months emerged. Since our analyses indicated that 83.2% of infants were rated by their parents as never or almost never exhibiting tactile sensory avoiding behaviours, it is possible that 10-month-old infants may not yet possess the ability to manifest active avoidance strategies. Seeking (as opposed to avoiding) may represent the preferred strategy of information prioritization in early infancy (Piccardi, Johnson, Gliga, 2020). Indeed, formal assessment of the ITSP internal consistency indicate that the avoiding quadrant has low internal consistency during the first two years of life (Cronbach σ = 0.56). Conversely, the seeking quadrant is reported to have adequate internal consistency (Cronbach σ = 0.79). Sensory avoiding behaviours may increase in frequency and improve in reliability over development, as children gain copying skills and active control over their sensory environment.

Associations between behavioural/neural markers and general development

Associations with behavioural markers. A significant positive association emerged in the whole sample between differential moving score (post S2 – pre S1) at 10 months and concurrent Mullen, R (87) = .238, p = .012, R^2 =.057. The association with later Mullen (24 months) trended towards significance, R (74) = .185, p = .055, R^2 = .034. These results indicate that infants manifesting more movement after receiving the tactile stimulation at 10 months also exhibited higher scores on the Mullen Scales at 10 and 24 months.

The association between differential looking score (post S2 – pre S1) and 10-month Mullen was not statistically significant, R (87) = -.065, p = .271, R^2 =.004; and 24-month Mullen was also not statistically significant, R (74) = .005, p = .483, R^2 =.000.

Associations with neural markers. There was no significant association between S1 alpha desynchronization and 10-month Mullen, R (88) = -.015, p = .443, R^2=.000; and 24-month Mullen, R (88) = .023, p = .420, R^2 =.000. The association between TSI and 10-month Mullen was statistically significant, R (88) = .177, p = .047, R^2 = .031; and 24-month Mullen was also statistically significant, R (88) = .210, p = .033, R^2 =.044. These results indicate that infants manifesting better neural repetition suppression of tactile stimulation at 10 months also exhibited higher scores on the Mullen Scales at 10 and 24 months.

This evidence suggests that enhanced neural repetition suppression may positively impact learning, both concurrently and longitudinally. It is possible that enhanced neural repetition suppression supports learning by speeding up priors updating (Pellicano & Burr, 2012). Previous evidence indicates that neural repetition suppression underlies efficient learning during experimental testing (León-Carrión et al., 2010). However, no studies attempted to assess the long-term effects of neural repetition suppression, especially through longitudinal designs. While in our study we measured neural repetition suppression in the tactile modality, we expect the link documented to manifest across sensory modalities. Future research should assess the potential links existing between neural repetition suppression in other sensory modalities (e.g. auditory) and both concurrent and longitudinal learning outcomes.

References

Charman T, Baird G, Simonoff E, Loucas T, Chandler S, Meldrum D, Pickles A. Efficacy of three screening instruments in the identification of autistic-spectrum disorders. Br J Psychiatry. 2007;191(6):554–9.

Conners CK. Conners third edition (Conners 3). Los Angeles, CA: Western Psychological Services; 2008.

Conners, C. K., & Goldstein, S. (2009). Conners Early childhood: Manual. Toronto, ON: Multi-Health Systems Incorporated.

Conners CK, Erhardt D, Sparrow E, Conners'Adult ADHD. Rating scales (CAARS). Toronto: MHS; 1999.

Constantino JN, Gruber CP. The social responsiveness scale. Los Angeles: Western Psychological Services; 2002.

Dunn, W. (2002). Infant/toddler sensory profile: User's manual. San Antonio, TX: Psychological Corporation

Evers, K., Debbaut, E., Maljaars, J., Steyaert, J., & Noens, I. (2020). Do Parental Interviews for ASD Converge with Clinical Diagnoses? An Empirical Comparison of the 3di and the DISCO in Children with ASD, a Clinically-Referred Group, and Typically Developing Children. Journal of Autism and Developmental Disorders, 1-13.

Goodman R, Ford T, Richards H, Gatward R, Meltzer H. The development and well-being assessment: description and initial validation of an integrated assessment of child and adolescent psychopathology. J Child Psychol Psychiatry. 2000;41(5):645–55.

León-Carrión J, Izzetoglu M, Izzetoglu K, Martín-Rodríguez JF, Damas-López J, y Martin JMB, Domínguez-Morales MR. Efficient learning produces spontaneous neural repetition suppression in prefrontal cortex. Behav Brain Res. 2010;208(2):502–8.

Lord C, Risi S, DiLavore PS, Shulman C, Thurm A, Pickles A. Autism from 2 to 9 years of age. Arch Gen Psychiatry. 2006;63(6):694–701.

Lord, C., Brugha, T. S., Charman, T., Cusack, J., Dumas, G., Frazier, T., ... & Taylor, J. L. (2020). Autism spectrum disorder. Nature reviews Disease primers, 6(1), 1-23.

Nobel E, Brunnekreef JA, Schachar RJ, van den Hoofdakker BJ, Hoekstra PJ. Parent–clinician agreement in rating the presence and severity of attention-deficit/hyperactivity disorder symptoms. ADHD Attention Deficit and Hyperactivity Disorders. 2019;11(1):21–9.

Pellicano, E., & Burr, D. (2012). When the world becomes 'too real': a Bayesian explanation of autistic perception. *Trends in cognitive sciences*, *16*(10), 504-510.

Piccardi, E. S., Johnson, M. H., & Gliga, T. (2020). Explaining individual differences in infant visual sensory seeking. Infancy, 25(5), 677-698.

Russell G, Rodgers LR, Ukoumunne OC, Ford T. Prevalence of parent-reported ASD and ADHD in the UK: findings from the Millennium Cohort Study. J Autism Dev Disord. 2014;44(1):31–40

Rutter M, Bailey A, Lord C. SCQ. The Social Communication Questionnaire. Torrance: Western Psychological Services; 2003.

Simonoff E, Pickles A, Charman T, Chandler S, Loucas T, Baird G. Psychiatric disorders in children with autism spectrum disorders: prevalence, comorbidity, and associated factors in a population-derived sample. J Am Acad Child Adolesc Psychiatry. 2008;47(8):921–9.

Tavakol M, Dennick R. Making sense of Cronbach's alpha. Int J Med Educ. 2011;2:53.

SM3 - Figure list

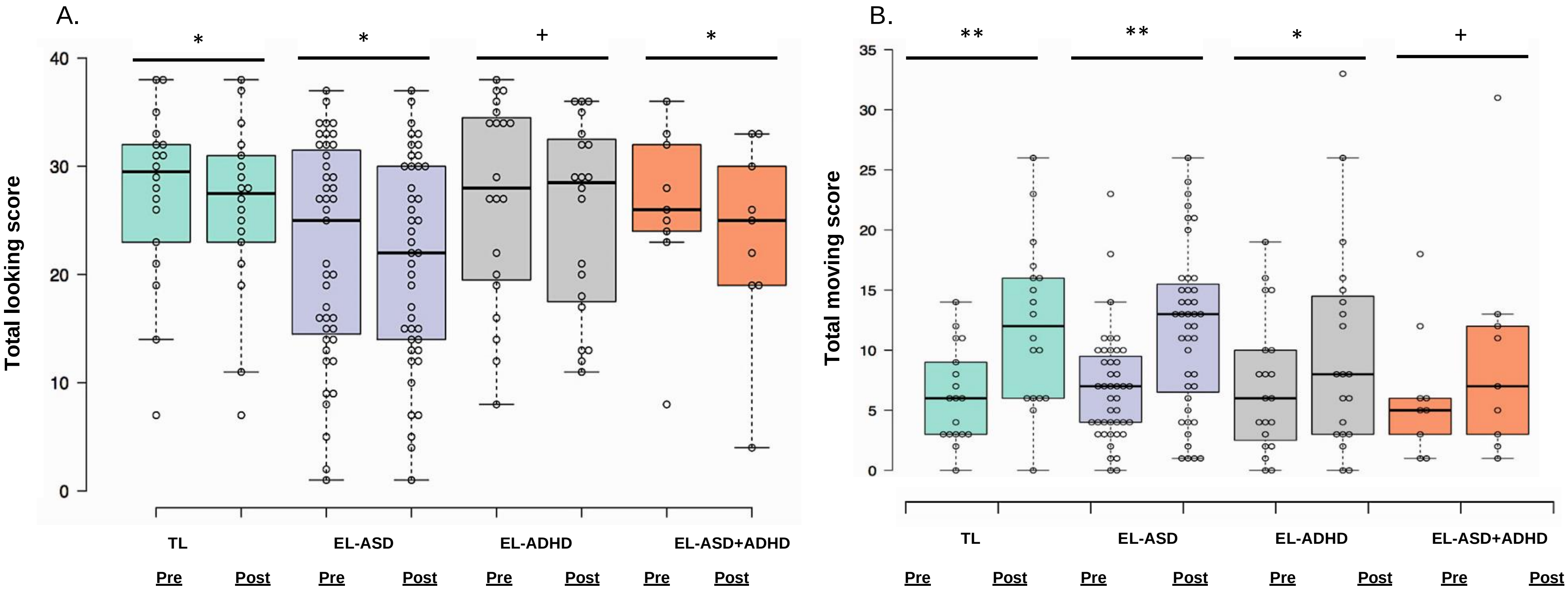

SM3 Figure 1. Boxplots illustrating A. Total looking score and B. Total moving score during the pre-stimulus phase (Pre-S1) and the post-stimulus phase (Post-S2) for each participant group (green=infants at typical likelihood of ASD or ADHD; violet=infants at elevated likelihood of ASD; grey=infants at elevated likelihood of ADHD; orange=infants at elevated likelihood of ASD and ADHD). *p<.05; **p<.001; +p=trending towards statistical significance

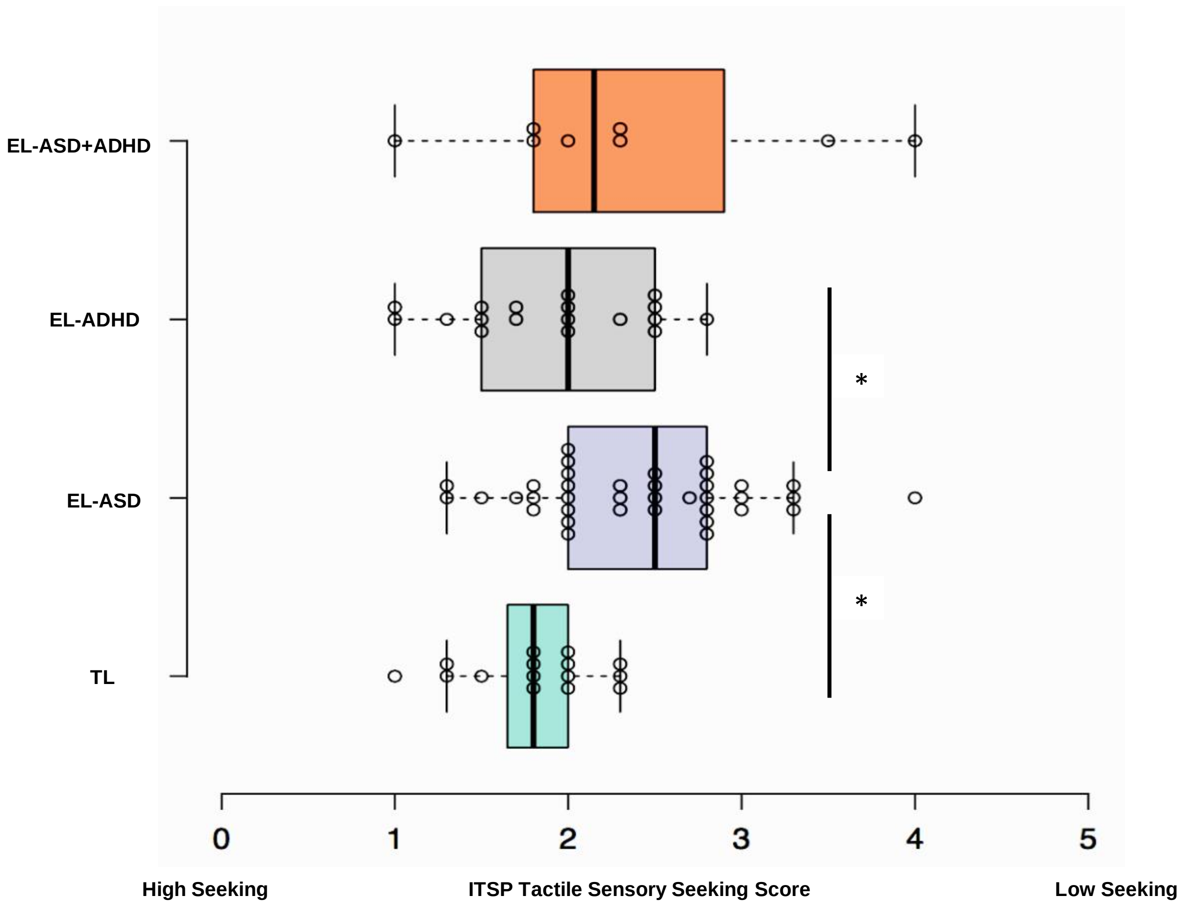

SM3 Figure 2. Boxplots illustrating the ITSP tactile sensory seeking scores at 10 months for infants contributing to the EEG analyses (green=infants at typical likelihood of ASD or ADHD; violet=infants at elevated likelihood of ASD; grey=infants at elevated likelihood of ADHD; orange=infants at elevated likelihood of ASD and ADHD).

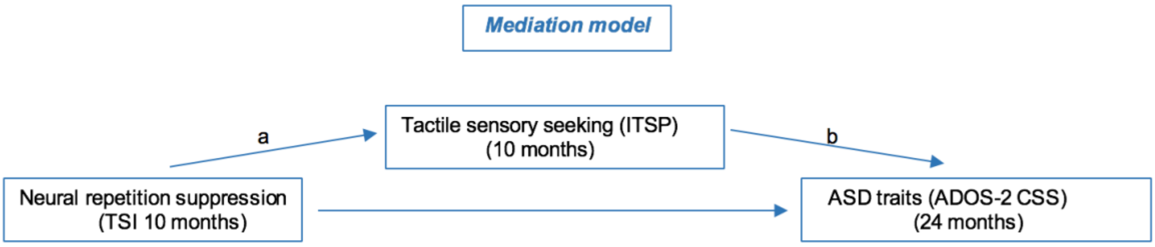

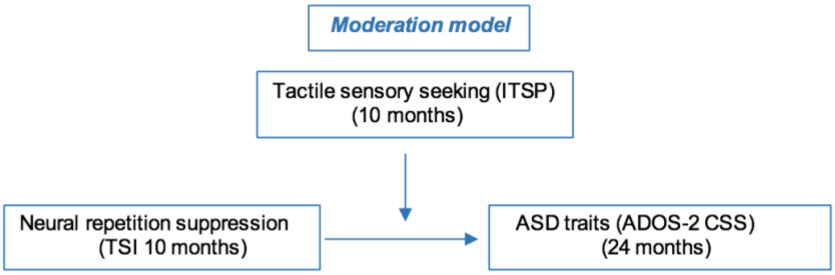

SM3 Figure 3. Mediation and moderation models illustrating the possible relationships between neural repetition suppression of tactile stimulation at 10 months, parent-reported tactile sensory seeking at 10 months and ASD traits at 24 months. The mediation model implies a causal relationship, whereby reduced neural repetition suppression leads to concurrent reduced tactile sensory seeking, which causes later elevated ASD traits. The moderation model implies an interaction effect whereby, at the same level of early neural repetition suppression of tactile stimulation, infants scoring high in concurrent tactile sensory seeking develop fewer ASD traits at 24 months.

SM4 - Table list

SM4 Table 1. Detailed characterisation of EL-ASD, EL-ADHD, EL-ASD+ADHD and TL infants who participated to the 10-month and 24-month visits

	EL-ASD	EL-ADHD	EL-ASD+ADHD	TL	*p values*
**10-month visit**					
Age in days	319.23 (14.68)	324.12 (27.75)	319.70 (14.66)	321.24 (17.17)	.684 (ns)
MSEL ELC	88.26 (15.04)	85.04 (15.61)	85.50 (3.79)	88.60 (12.62)	.713 (ns)
MSEL GM	38.45 (9.59)	39.00 (10.22)	35.75 (10.17)	35.32 (12.00)	.409 (ns)
MSEL FM	50.61 (11.31)	51.92 (13.96)	49.60 (12.41)	50.64 (12.67)	.934 (ns)
MSEL VR	49.91 (9.42)	47.04 (9.80)	48.25 (7.73)	48.92 (8.19)	.554 (ns)
MSEL RL	37.92 (10.55)	35.04 (10.22)	35.35 (10.92)	40.00 (8.90)	.271 (ns)
MSEL EL	36.67 (12.84)	34.38 (12.10)	36.05 (15.33)	36.40 (10.13)	.887 (ns)
N (% boys)	79 (51.9)	27 (55.6)	21 (57.1)	25 (56)	
ITSP Tactile Seeking	2.38 (0.62)$_a$	1.90 (0.54)	2.33 (0.97)	1.81 (0.39)	.005*
	EL-ASD	EL-ADHD	EL-ASD+ADHD	TL	*p values*
**24-month visit**					
Age in days	774.90 (48.00)	766.43 (37.65)	756.56 (22.65)	762.25 (36.07)	.343 (ns)
MSEL ELC	101.40 (20.01)$_a$	107.00 (21.72)	96.94 (17.12)$_a$	114.25 (17.90)	.020*
MSEL GM	N/A	N/A	N/A	N/A	
MSEL FM	50.34 (11.14)	52.19 (11.90)	51.75 (10.93)	56.00 (12.98)	.253 (ns)
MSEL VR	49.34 (12.79)$_a$	56.71 (12.04)	47.94 (10.28)$_a$	56.62 (10.66)	.001**
MSEL RL	51.46 (13.55)	52.24 (14.12)	49.00 (10.39)	57.67 (8.73)	.128 (ns)
MSEL EL	50.16 (14.70)	52.52 (14.07)	44.94 (11.07)	55.42 (12.30)	.114 (ns)
N (% boys)	62 (50)	21 (44.4)	16 (52.4)	24 (48.1)	
ADOS-2 CSS	2.83 (2.17)$_a$	2.75 (2.09)	3.69 (0.57)$_a$	1.55 (0.67)	.004*
ECBQ Inhibitory Control	3.67 (1.19)	3.80 (0.88)	3.61 (1.28)	4.095 (0.96)	.632 (ns)
ECBQ Activity	4.69 (0.86)	5.06 (1.04)	5.19 (0.94)	4.76 (0.74)	.159 (ns)
Q-CHAT	24.41 (11.61)	28.47 (10.63)	28.51 (12.63)	20.68 (5.48)	.120 (ns)

* $p < .05$

** $p \leq .001$

$_a$ indicates significant differences with the TL group

M (*SD*) reported for: Age in days; MSEL ELC = Mullen Scales for Early Learning Early Composite Score; MSEL GM = Mullen Scales for Early Learning Gross Motor Score; MSEL FM = Mullen Scales for Early Learning Fine Motor Score; MSEL VR = Mullen Scales for Early Learning Visual reception Score; MSEL RL = Mullen Scales for Early Learning Receptive Language Score; MSEL EL = Mullen Scales for Early Learning Expressive Language; ADOS-2 CSS = ADOS-2 Calibrated Severity Scores; ITSP Tactile Seeking = Tactile sensory seeking average score of the Infant-Toddler Sensory Profile; ECBQ Inhibitory Control = Inhibitory Control subscale of the Early Childhood Behaviour Questionnaire; ECBQ Activity = Activity subscale of the Early Childhood Behaviour Questionnaire; Q-CHAT = Quantitative Checklist for Autism in Toddlers.

SM4 Table 2. Detailed comparison of participants included and excluded from the EEG analyses due to fussiness/excessive movement artifacts on behavioural assessment scales at 10 and 24 months

	Excluded	Included	*p values*
**10-month visit**			
Age in days	322.06 (15.97)	321.11 (18.92)	.796 (ns)
MSEL ELC	85.91 (13.36)	86.73 (14.41)	.773 (ns)
MSEL GM	35.47 (9.66)	36.81 (9.41)	.484 (ns)
MSEL FM	48.73 (12.11)	50.69 (11.92)	.419 (ns)
MSEL VR	47.44 (8.74)	48.59 (8.73)	.515 (ns)
MSEL RL	38.00 (10.19)	36.68 (9.95)	.510 (ns)
MSEL EL	36.02 (11.76)	35.97 (12.53)	.983 (ns)
N (% boys)	34 (56)	91 (49.5)	
ITSP Tactile Seeking	4.38 (0.80)	4.38 (0.64)	.979 (ns)

	Excluded	Included	*p values*
**24-month visit**			
Age in days	766.74 (44.31)	771.30 (44.48)	.648 (ns)
MSEL ELC	103.48 (18.39)	105.22 (21.06)	.704 (ns)
MSEL GM	N/A	N/A	N/A
MSEL FM	51.63 (11.94)	52.75 (11.51)	.665 (ns)
MSEL VR	51.74 (12.82)	53.34 (13.04)	.581 (ns)
MSEL RL	53.92 (11.86)	52.06 (12.72)	.506 (ns)
MSEL EL	49.67 (10.40)	51.23 (15.01)	.618 (ns)
N (% boys)	27 (53)	77 (40)	
ADOS-2 CSS	2.69 (2.20)	2.62 (2.03)	.878 (ns)
ECBQ Inhibitory Control	3.54 (0.94)	3.83 (1.20)	.278 (ns)
ECBQ Activity	5.02 (0.99)	4.68 (0.85)	.110 (ns)
Q-CHAT	23.59 (9.13)	25.02 (11.27)	.583 (ns)

M (*SD*) reported for: Age in days; MSEL ELC = Mullen Scales for Early Learning Early Composite Score; MSEL GM = Mullen Scales for Early Learning Gross Motor Score; MSEL FM = Mullen Scales for Early Learning Fine Motor Score; MSEL VR = Mullen Scales for Early Learning Visual reception Score; MSEL RL = Mullen Scales for Early Learning Receptive Language Score; MSEL EL = Mullen Scales for Early Learning Expressive Language; ADOS-2 CSS = ADOS-2 Calibrated Severity Scores; ITSP Tactile Seeking = Tactile sensory seeking average score of the Infant-Toddler Sensory Profile; ECBQ Inhibitory Control = Inhibitory Control subscale of the Early Childhood Behaviour Questionnaire; ECBQ Activity = Activity subscale of the Early Childhood Behaviour Questionnaire; Q-CHAT = Quantitative Checklist for Autism in Toddlers.

Appendix A

EU AIMS Medical and Psychiatric History v3 17.9.2013

Site __________ ID______________________Date: _____/_____/__ __ __ __Interviewer__________
Relationship of responder to child to be seen in study (circle one):
biological mother stepmother adoptive mother
biological father stepfather adoptive father Other (please explain)___________

3. The items below ask about the medical history of the child participating in the research project (and that of the child's *blood* relatives). In the first column, please indicate anyone in the family has the given disorder or problem listed (No, Yes, or Not Sure). If you select Yes or Not Sure, write Y (yes) or NS (not sure) under the person/persons witl that disorder/problem. All family relationships refer to the child participating in the research project (e.g. Siblings = Siblings of child participating in research project).

	(circle one for each item)			*Only complete detail information if child or child's blood relative has disorder or problem*													
Disorder or Problem	***Child/blood relative has disorder***			***IF Y or NS***	***Child***	***Parents of Child***		***Siblings***	***½ Siblings***		***First Cousins***		***Aunts & Uncles***		***Grand-parents***		***Other***
	NO	***YES***	***NOT SURE***	→		*Mom*	*Dad*	*N/A*	*Mat*	*Pat*	*Mat*	*Pat*	*Mat*	*Pat*	*Mat*	*Pat*	*Specify Relationship*
a. Autism Spectrum Disorder	N	Y	NS														
b. Fragile X	N	Y	NS														
c. Tuberous Sclerosis	N	Y	NS														
d. Neurofibromatosis	N	Y	NS														
e. Rett Syndrome	N	Y	NS														
f. Childhood Disintegration Disorder	N	Y	NS														
g. Prader Willi Syndrome	N	Y	NS														
h. Angelman Syndrome	N	Y	NS														
i. Other chromosomal abnormality, disorder or syndrome ______ *(please specify)*	N	Y	NS														
j. Congenital rubella	N	Y	NS														
k. PKU	N	Y	NS														
l. Hydrocephalus (water on the brain)	N<br>N	Y<br>Y	NS<br>NS														
m. Cerebral Palsy	N	Y	NS														
n. Intellectual Disability	N	Y	NS														
o. Seizures	N	Y	NS														
p. Speech delay requiring therapy	N	Y	NS														
q. Attention Deficit Disorder (ADHD)	N	Y	NS														
r. Panic or anxiety disorder	N	Y	NS														
s. Depression	N	Y	NS														
t. Manic depression/Bipolar	N	Y	NS														
u. Schizophrenia	N	Y	NS														
v. Been admitted to a hospital for psychiatric illness	N	Y	NS														
w. Birth defects (e.g., cleft lip or palate, open spine)	N	Y	NS														
y. Norrie Syndrome……………..	N	Y	NS														
